# Immunocapture of dsRNA-bound proteins provides insight into *Tobacco rattle virus* replication complexes and reveals Arabidopsis DRB2 to be a wide-spectrum antiviral effector

**DOI:** 10.1093/plcell/koab214

**Published:** 2021-08-26

**Authors:** Marco Incarbone, Marion Clavel, Baptiste Monsion, Lauriane Kuhn, Hélène Scheer, Émilie Vantard, Vianney Poignavent, Patrice Dunoyer, Pascal Genschik, Christophe Ritzenthaler

**Affiliations:** 1 Institut de Biologie Moléculaire des Plantes, CNRS, Université de Strasbourg, 67000 Strasbourg, France; 2 Plateforme Protéomique Strasbourg Esplanade FR1589 du CNRS, Université de Strasbourg, Strasbourg, France

## Abstract

Plant RNA viruses form organized membrane-bound replication complexes to replicate their genomes. This process requires virus- and host-encoded proteins and leads to the production of double-stranded RNA (dsRNA) replication intermediates. Here, we describe the use of *Arabidopsis thaliana* expressing GFP-tagged dsRNA-binding protein (B2:GFP) to pull down dsRNA and associated proteins *in planta* upon infection with *Tobacco rattle virus* (TRV). Mass spectrometry analysis of the dsRNA-B2:GFP-bound proteins from infected plants revealed the presence of viral proteins and numerous host proteins. Among a selection of nine host candidate proteins, eight showed relocalization upon infection, and seven of these colocalized with B2-labeled TRV replication complexes. Infection of *A. thaliana* T-DNA mutant lines for eight such factors revealed that genetic knockout of *dsRNA-BINDING PROTEIN 2* (*DRB2*) leads to increased TRV accumulation and *DRB2* overexpression caused a decrease in the accumulation of four different plant RNA viruses, indicating that DRB2 has a potent and wide-ranging antiviral activity. We propose B2:GFP-mediated pull down of dsRNA to be a versatile method to explore virus replication complex proteomes and to discover key host virus replication factors. Given the universality of dsRNA, development of this tool holds great potential to investigate RNA viruses in other host organisms.

## Teaser

Double-stranded RNA immunoprecipitation allows the identification of Arabidopsis host proteins associated with tobacco rattle virus replication complexes.

##  

**Figure:**
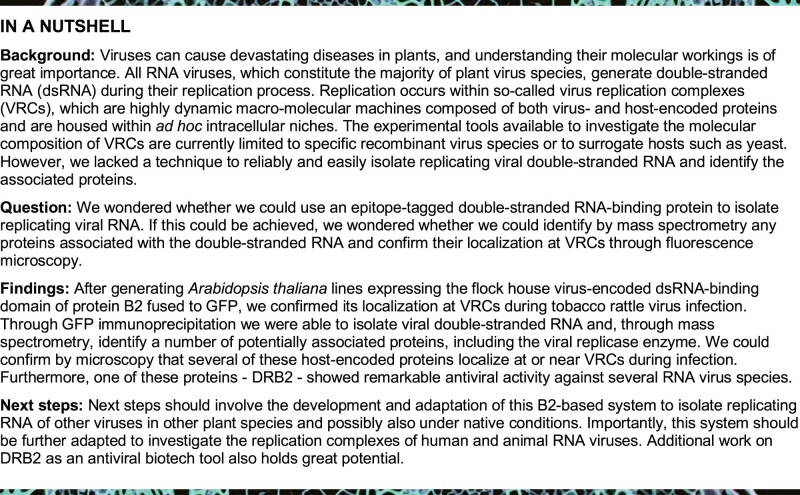


## Introduction

Viruses are obligate endocellular parasites that hijack their host’s molecular processes and machinery to multiply, a process that sometimes results in devastating diseases. Pivotal to a successful infection is the efficient replication of the viral genomic nucleic acid(s). In the majority of plant virus species, the genome consists of one or more molecules of single-stranded positive polarity RNA, or (+)ssRNA. Replication is carried out by the virus-encoded RNA-dependent RNA-polymerase (RdRp), often part of a larger protein known as the replicase. This enzyme first copies the viral (+) genome into (-)ssRNA that is then used as a template for the production of progeny (+)ssRNA. Intrinsic to the RNA replication process is the generation of long double-stranded RNA (dsRNA) intermediates by the viral RdRp.

The replication of all known (+) strand RNA virus takes place on host membranes whose origin, whether the endoplasmic reticulum, chloroplasts, mitochondria, peroxisomes, etc., depends on virus species (reviewed in Ritzenthaler and Elamawi, 2006; Verchot, 2011; Grangeon et al., 2012; Jiang and Laliberté, 2016). The progressive virus-induced recruitment of such membranes generally leads to dramatic reorganizations of the host endomembrane system into so called “viral factories.” These viral factories are the sites where all steps vital to the virus life are carried out including protein translation, RNA encapsidation, and RNA replication *sensu stricto*.

The specialized molecular entities on which RNA replication occurs within the viral factories are known as the virus replication complexes (VRCs). While a minimal VRC arguably consists of single- and double-stranded viral RNA and replicase, their precise composition, which depends on the virus and host species, remains largely unexplored. A number of studies have shown that specific host proteins can be integral part of these VRC and exert positive (pro-viral) or negative (anti-viral) effects on replication. Our knowledge on these host proteins is summarized in several exhaustive reviews (Nagy and Pogany, 2011; Wang, 2015; Nagy, 2016). These include among others RNA-binding proteins, RNA helicases, chaperones, and proteins belonging to the RNA interference machinery (RNAi, or RNA silencing), which is the primary response to replicating viruses in plants and other eukaryotes (Wang, 2015; Barton et al., 2017).

Antiviral RNAi against RNA viruses in the model plant *Arabidopsis thaliana* is initiated by RNAse-III Dicer-Like enzymes DCL4 and DCL2, which cleave dsRNA into 21- and 22-nt small-interfering RNA (siRNA), respectively. These siRNAs are then loaded into Argonaute (AGO) proteins, which use them as templates to recognize and cleave viral ssRNA in a sequence-specific manner. Viruses have evolved a vast array of strategies to evade or block RNAi, the best studied of which are viral suppressors of RNA silencing (VSRs). These proteins suppress silencing through a wide range of molecular strategies, from inhibition of dicing, to siRNA sequestration, to AGO degradation (Incarbone and Dunoyer, 2013). Of note, *A. thaliana* encodes several RNAse-III-like enzymes (RTLs) in addition to Dicers, but little is known regarding their function (Elvira-Matelot et al., 2016). The accumulation of viral dsRNA in planta varies widely among (+)ssRNA virus and host species, as we have recently shown (Monsion et al., 2018). While the precise molecular events occurring during virus RNA replication are often unclear, it can be argued that rapid and efficient separation of the (+) and (-) RNA strands could not only allow more replication cycles to take place, but also constitute a powerful mechanism of RNA silencing evasion/suppression through removal of dsRNA.

A great deal of our knowledge on plant virus VRCs emerged from a series of seminal studies conducted with *Tomato bushy stunt virus* (TBSV) on yeast (*Saccharomyces cerevisiae*), a surrogate host used as a powerful biological tool to conduct genetic screens and functional studies on host factors involved in TBSV VRC activity (reviewed in Nagy et al., 2016). While the authors of these studies, where possible, validated the results obtained in yeast and in vitro in *Nicotiana benthamiana*, data obtained in planta on VRCs of other viruses remains sparse. Recent studies have reported methods to spatiotemporally visualize VRCs in vivo via fluorescently labeled dsRNA-binding proteins (Cheng et al., 2015; Barton et al., 2017; Monsion et al., 2018). Candidate-based reverse genetic approaches have been used to probe the involvement of host factors in VRC formation and activity in plants (Wei et al., 2013; Li et al., 2016). These approaches, however, are necessarily based on prior discovery acquired by other experimental means. Another experimental strategy successfully used to characterize VRCs has been to pull down tagged viral proteins and analyze the resulting protein populations by mass spectrometry (MS; Dufresne et al., 2008; Lohmus et al., 2016; Wang et al., 2018). While this last method has provided valid and compelling data, we decided to investigate VRCs from a viral RNA-centered, rather than viral protein-centered, perspective. We hypothesized that pull-down of dsRNA from virus-infected plants followed by MS of dsRNA-associated proteins would provide insight into the molecular composition of VRCs.

In this study, we report that the dsRNA-binding domain of FHV protein B2, when fused to GFP (henceforth, B2:GFP) can not only be used to visualize VRCs in planta as shown previously in *N. benthamiana* (Monsion et al., 2018), but additionally can be exploited as a means to pull-down dsRNA-associated nucleoprotein complexes from infected Arabidopsis plants. As a proof of concept, we used *Tobacco rattle virus* (TRV genus: *Tobravirus*, family: *Virgaviridae*), a well-studied (+)ssRNA virus, to perform B2:GFP immunoprecipitations (IPs). In this manner, a number of virus- and host-encoded proteins could be identified and validated for their localization in and around VRCs. Among these candidates, dsRNA-binding protein (DRB2), a dsRNA-binding protein was found to display antiviral activity by using loss- and gain-of-function approaches. These results provide robust validation of dsRNA pull-down as an effective and high-throughput method for VRC characterization in planta. Furthermore, the results offer detailed snapshots of TRV replication complexes and viral factories, with host factors showing unique and distinct localization patterns in and around these complexes.

## Results

### B2:GFP-mediated isolation of TRV dsRNA from *A. thaliana*

The dsRNA-binding B2:GFP protein, when ectopically expressed in transgenic *N. benthamiana*, has been previously shown to specifically associate with the VRCs of several positive-strand RNA viruses from plants and insects (Monsion et al., 2018). For the sake of clarity, VRCs refers hereafter to all the factors that directly and/or indirectly associate to the viral replicating dsRNA rather than to the replicase core complex *sensu strito*. Following these findings, we wished to further exploit B2:GFP as a biochemical bait to explore the composition and biology of RNA VRCs, the pivotal element of which is dsRNA. To do so, and given the versatility of *A. thaliana* as a model plant species, we first produced homozygous 35S:*B2:GFP* transgenic plants. Although in this work we focused essentially on the 35S:*B2:GFP/*Col-0 line, 35S:*B2:GFP* was also introduced into various genetic backgrounds including mutants of the core antiviral Dicer-Like genes, *dcl2-1*, *dcl4-2*, and triple *dcl2-1/dcl3-1/dcl4-2* (Supplemental Figure S1, A–C). The rationale behind this choice is that DCL proteins are arguably the best-known RNAse III enzymes in plants, and the siRNA they generate from virus-derived dsRNA precursors are the effectors of RNA silencing, the main antiviral defense in plants (Incarbone and Dunoyer, 2013; Pumplin and Voinnet, 2013).

Similar to the B2:FP *N. benthamiana* lines (Monsion et al., 2018) and despite the clear expression of B2:GFP (Supplemental Figure S1A), the transgenic lines in the different *dcl* mutant backgrounds showed little to moderate developmental phenotypes (Supplemental Figure S1C). These were reminiscent of (but distinct from) those caused by ectopic expression of other RNA silencing suppressors such as P19 or HC-Pro (Kasschau et al., 2003; Incarbone et al., 2017). In addition to leaf serration, the elongated and slightly downward-curled leaves of the B2:GFP line are reminiscent of mutants in the miR390/TAS3 pathway such as *rdr6*, *dcl4*, and *ago7* (Adenot et al., 2006). Such phenotypes may be determined (1) by inhibition of long dsRNA processing into siRNA and/or (2) by disruption of miRNA function through their sequestration.

The full-length FHV B2 protein has been shown to be a suppressor of RNA silencing (Li et al., 2002; Seo et al., 2012), and proposed to act through both inhibition of dicing and sequestration of siRNA duplexes (Chao et al., 2005). To investigate whether the GFP-tagged 73 amino acid dsRNA binding domain of B2 that lacks the residues involved in the interaction with PAZ domains of Dicer proteins (Singh et al., 2009; Liu et al., 2012) also acts as a suppressor of RNA silencing, we performed a standard GFP silencing patch test on *N. benthamiana* leaves (Supplemental Figure S1, D and E). B2, as a C-terminal fusion to tRFP (B2:tRFP) was able to suppress silencing of the GFP transgene, as was turnip crinkle virus suppressor P38. By contrast, a C44S, K47A double-mutated version of B2:tRFP impaired in dsRNA binding (Monsion et al., 2018) was unable to suppress silencing, suggesting that suppression activity is dsRNA-binding-dependent and likely DCL-binding-independent.

Next, we investigated the effects of stably expressed B2:GFP on endogenous small RNA pathways: biogenesis of microRNAs 159 and 160 was not perturbed in 35S:*B2:GFP*/Col-0 plants, while biogenesis of siRNA such as endo-siRNA (IR71) and trans-acting siRNA (TAS1) was completely abolished (Supplemental Figure S1B). Whether these defects in endo-siRNA and trans-acting siRNA biogenesis are responsible of the observed developmental phenotypes remains to be determined.

**Figure 1:**
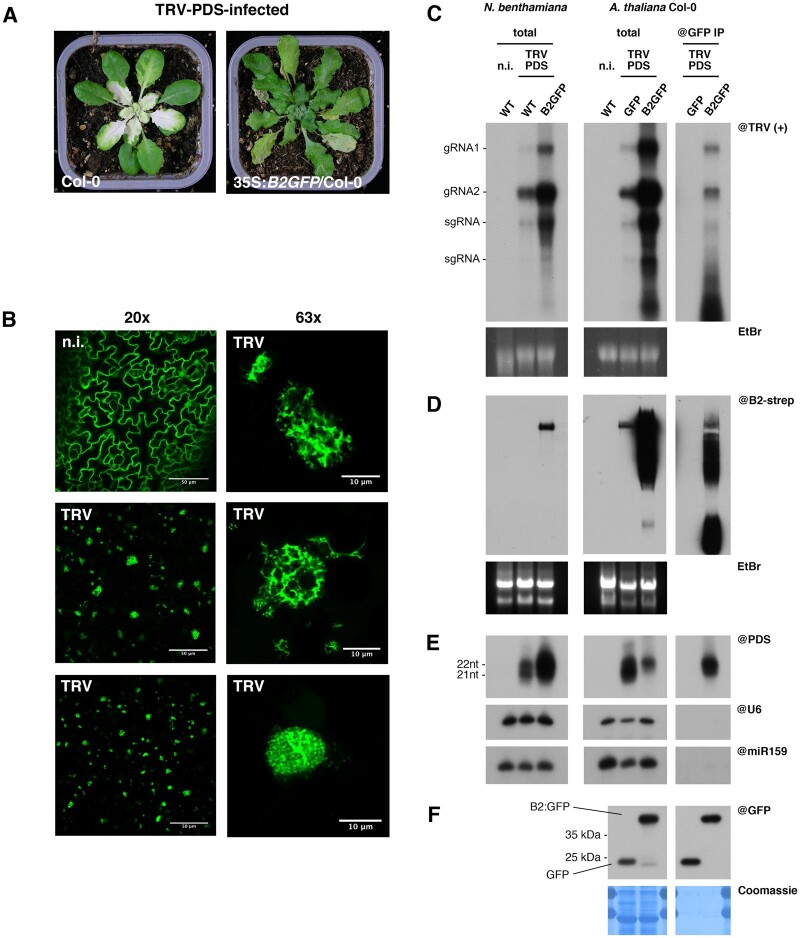
IP of B2:GFP allows the isolation of TRV dsRNA in vivo. A, Photos of *A. thaliana* Col-0 and 35S:*B2:GFP*/Col-0 plants 13 dpi with TRV-PDS. B, Confocal microscopy analysis of noninfected (n.i., top left) and TRV-PDS systemically infected leaves of 35S:*B2:GFP*/Col-0 plants. On the right, higher magnification (×63) images of TRV replication complexes from the same tissues as those visible at lower magnification (×20) on the left middle and bottom. C, RNA gel blot analysis of high molecular weight RNA from total fractions of TRV-PDS-infected wild-type or 35S:*B2:GFP N. benthamiana* (left), and from total (middle) and anti-GFP immunoprecipitated (right) fractions from infected 35S:*GFP* and 35S:*B2:GFP/*Col-0 *A. thaliana*. D, DsRNA–protein gel blot analysis of native-state high molecular weight RNA from samples described in (C). EtBr staining was used as loading control in (C) and (D). Note the absence of detectable levels of dsRNA species in the noninfected WT plants in agreement with previous similar analyses (Monsion et al., 2018). E, RNA gel blot analysis of low molecular weight RNA from samples described in (C). The probes were applied sequentially on the same membrane in successive rounds of probing and stripping. snU6 and miR159 were used as loading controls. F, Immunoblot analysis of proteins from the same IP experiment analyzed in (C).

We then infected 35S:*B2:GFP*/Col-0 plants with a recombinant TRV carrying part of the *PHYTOENE DESATURASE* (*PDS*) gene (Liu et al., 2002). As expected, the control Col-0 plants showed minor viral symptoms and the typical bleaching phenotype linked to PDS gene silencing. In contrast, the B2:GFP-expressing plants showed no significant leaf discoloration but severe viral symptoms (Figure 1A) and death of the plants occurred before flowering (not shown), well in agreement with the efficient RNA silencing suppression activity of the B2 dsRNA-binding domain. Observation of systemically infected leaves by confocal microscopy showed TRV-induced relocalization of B2:GFP to distinct cytosolic mesh-like structures (Figure 1B) reminiscent to those observed in 35S:*B2:GFP*/*N. benthamiana* and shown to correspond to TRV-induced VRCs (Monsion et al., 2018). RNA gel blot analysis of RNA from TRV-PDS systemically infected 35S:*B2:GFP*/*N. benthamiana* and 35S:*B2:GFP*/Col-0 revealed that B2:GFP caused a striking over-accumulation of viral (+)ssRNA in both plant species (Figure 1C). This is also in agreement with B2:GFP activity as a suppressor of RNA silencing, and could be recapitulated in a *dcl2-1 dcl4-2* double mutant (*dcl24*), which lacks the two main antiviral Dicers (Supplemental Figure S1F; Deleris et al., 2006). B2:GFP also caused a tremendous increase in long dsRNA content, likely corresponding to replication intermediates, as determined by dsRNA–protein gel blot (northwestern) blotting in both B2:GFP-expressing Col-0 and *N. benthamiana* plants (Figure 1D).

In contrast, virus-derived siRNAs (vsiRNA) accumulated differentially between Arabidopsis and *N. benthamiana*. Thus, while B2:GFP expression led to an overall reduction in 21 and 22 nt vsiRNA species in Col-0 plants, the opposite effect was observed in *N. benthamiana* (Figure 1E). Conversely, miR159 and U6-derived small nucleolar RNA (snRNA) accumulation was unaffected by the presence of B2:GFP in both plant species (Figure 1E;Supplemental Figure S1B). These results suggest that B2:GFP interferes with TRV RNA processing by DCL enzymes either by promoting (*N. benthamiana*) or preventing (Arabidopsis) siRNA production, in a manner that is associated with enhanced viral replication in both plant species. Whether increased TRV replication is a cause or a consequence in siRNA levels is unknown.

As a first experiment establishing B2:GFP as a tool to study VRC composition, we performed anti-GFP IPs from TRV-PDS-infected 35S:*B2:GFP*/Col-0 plants and analyzed their composition in (+)strand viral RNA (Figure 1C), long dsRNA (Figure 1D), siRNA (Figure 1E), and proteins (Figure 1F). As a negative control, we included TRV-PDS-infected 35S:*GFP*/Col-0 plants. RNA gel blot and dsRNA–protein gel blot analyses performed on IPed RNA revealed that immune complexes contained (+)ssRNA, long dsRNA and 22nt vsiRNA, but no U6 and miR159 (Figure 1, C–E). Interestingly, and in contrast with our previous report in vitro (Monsion et al., 2018) but well in agreement with the capacity of B2 to bind dsRNAs longer than 18 bp (Chao et al., 2005), antiviral siRNA were immunoprecipitated (Figure 1E). Immunoblot analysis of proteins from the same experiment revealed efficient IP of both GFP and B2:GFP (Figure 1F). Altogether we concluded that IP allowed the isolation of TRV double-stranded replication intermediates, the core element of VRCs.

### IP and identification of B2:GFP-associated viral and host proteins by MS

Once established that IP of B2:GFP from plants allowed efficient isolation of virus replication dsRNA intermediates, we wondered whether these complexes contain specific virus- and host-encoded proteins. To address this question, we performed anti-GFP IP on TRV-PDS-infected 35S:*GFP*/Col-0 versus 35S:*B2:GFP*/Col-0 in triplicate, and analyzed the immunoprecipitated proteins by MS. The complete list of identified viral and host proteins from this analysis is shown in Supplemental Data Set S1. We also performed the same IP and MS analysis on noninfected 35S:*GFP*/Col-0 versus 35S:*B2:GFP*/Col-0 plants (Supplemental Data Set S2).

**Figure 2:**
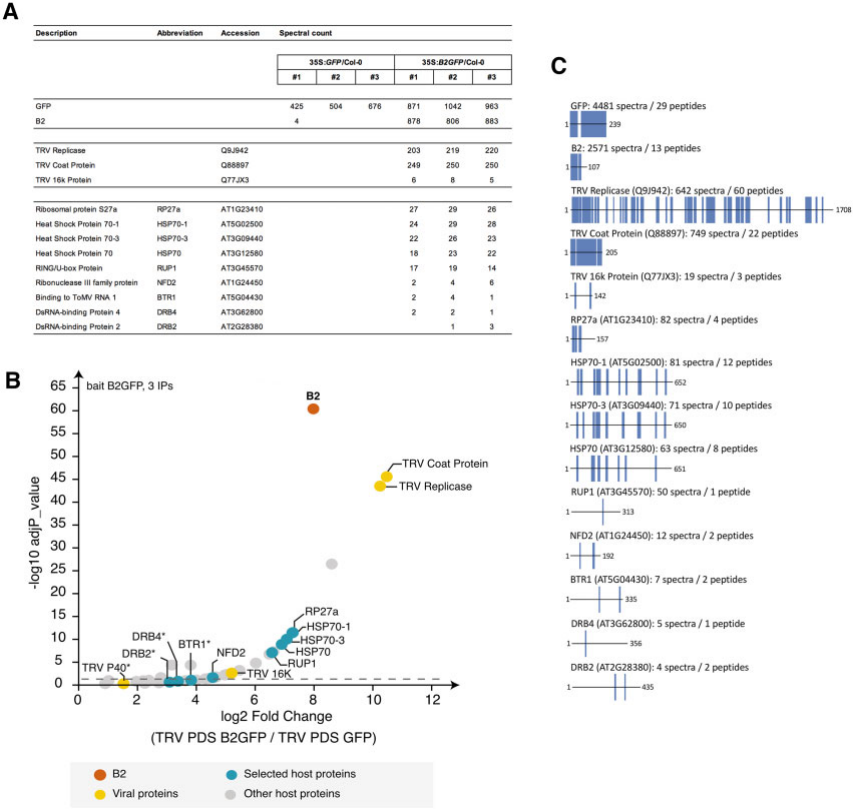
IP of B2:GFP allows the isolation of TRV proteins and host factors. MS analyses of anti-GFP immunoprecipitated proteins from TRV-PDS-infected 35S:GFP/Col-0 and 35S:B2:GFP/Col-0 plants. Three technical replicates were performed and analyzed per genotype. *Arabidopsis thaliana* proteins shown here are the ones that have been tested in this study. Abbreviations correspond to those available in the literature, except for RP27a and RUP1, which have been here assigned for lack of previously established ones. Accession numbers correspond to UniProt (TRV proteins) and TAIR (*A. thaliana* proteins) databases. A, Table containing the spectral counts obtained per technical replicate for bait proteins (GFP + B2), TRV proteins and *A. thaliana* proteins. B, Volcano plot representation shows the enrichment of proteins from TRV-infected plants that copurified with B2:GFP. *Y*- and *X*-axis display adjusted *P*-values and fold changes, respectively. The dashed line indicates the threshold above which proteins are significantly enriched (adjP < 0.05). Selected proteins below significance level are indicated with a star. The source data are available in Supplemental Data Sets 1 and 2. C, Sequence coverage obtained on all the proteins listed in (A). For each of the 14 entries, the length of the protein is displayed while the covered residues are highlighted with blue vertical bars. The total number of spectra matching on each protein in the three replicates is indicated, as well as the corresponding total number of unique peptide sequences. The complete protein list can be found in Supplemental Data Sets 1 and 2.

A preliminary analysis by immunoblot confirmed efficient and reproducible B2:GFP and GFP IP (Supplemental Figure S2), which could be confirmed by MS, reads from B2:GFP and GFP being the most abundant (Figure 2, A and B; Supplemental Data Set S1). We next searched and ranked accessions that were identified only—or enriched—in TRV-infected 35S:*B2:GFP*/Col-0 samples (Figure 2, A and B; Supplemental Data Set S1). As expected, the TRV replicase (Uniprot accession Q9J942) was among the most abundant proteins detected in IPs from B2:GFP samples (Figure 2). This result, along with the previously described detection of viral dsRNA in analogous IPs (Figure 1), suggests that B2:GFP IP allows the isolation of TRV VRCs. Detection of TRV coat protein (CP, Uniprot Q88897) and 16k suppressor of silencing (Uniprot Q77JX3) in B2:GFP IPs (Figure 2) also suggests that these viral proteins, not known to participate in replication, associate directly or indirectly to dsRNA. Although unlikely, we cannot at this point rule out that one or more of these TRV proteins bind B2:GFP and not dsRNA.

In addition to TRV-encoded proteins, MS analysis allowed also the identification of 110 host proteins exclusively present in IPs from B2:GFP-expressing plants (Figure 2; Supplemental Data Set S1), which we considered as replication complex-associated host protein candidates. Overall, 29 of these proteins were significantly enriched in the IPs with an adjusted *P*-value < 0.05 (Figure 2B). Volcano plots generated by the comparison of the TRV-infected 35S:*B2:GFP*/Col-0 samples with combinations of all controls (noninfected 35S:*B2:GFP*/Col-0, noninfected 35S:*GFP*/Col-0 and TRV-infected 35S: *GFP*/Col-0) are displayed in Supplemental Figure S3.

To evaluate the association of candidate host proteins to replication complexes and considering their high number (Supplemental Data Set S1), we arbitrarily restricted our analysis to a set of nine *A. thaliana* gene products that were either detected with high (>60) spectral counts (AT5G02500, AT3G09440, AT3G12580) or confirmed/potential RNA-binding/interacting proteins from literature or NCBI annotation (AT1G24450 and AT5G04430 [Fujisaki and Ishikawa, 2008]; AT3G62800 and AT2G28380 [Clavel et al., 2015]), or both (AT1G23410 and AT3G45570; Figure 2, A and B). The distribution of peptide reads along these selected proteins, along with the TRV and bait proteins, is shown in Figure 2C. It should be noted that four of the candidates (AT1G23410, AT3G09440, AT5G02500, and AT3G12580) were also present in B2:GFP IPs from noninfected plants (Supplemental Data Set S2) which may reflect their dsRNA binding activity in both healthy and virus-infected plants. However, the spectral count of peptides from these proteins was a fraction of that detected in IPs from TRV-infected plants, despite the spectral counts of the bait proteins being comparable. All other candidates were not detected in B2 IPs from noninfected plants. Finally, we excluded from our priority list a number of proteins that were significantly enriched in B2:GFP versus GFP plants due to the number of candidates to analyze and their apparent lack of significance in viral replication process based on literature. This includes, for instance, the most enriched protein, a myrosinase with anti-microbial activity that is present in Brassica crops and involved in defense against herbivores (Bhat and Vyas, 2019).

### tRFP does not label TRV replication complexes in planta

In a second step, we tested the subcellular localization of the selected candidates in relation to B2:GFP in healthy and TRV-infected plants. To do so, we opted for the 35S-driven transient expression of the Arabidopsis candidates as N- or C-terminal fusions to tRFP in healthy or TRV-infected 35S:*B2:GFP*/*N. benthamiana*. In all cases, confocal imaging was performed 3–4 days post agroinfiltration, a time that was found to be optimal for TRV-infection and transient expression of protein candidates.

**Figure 3:**
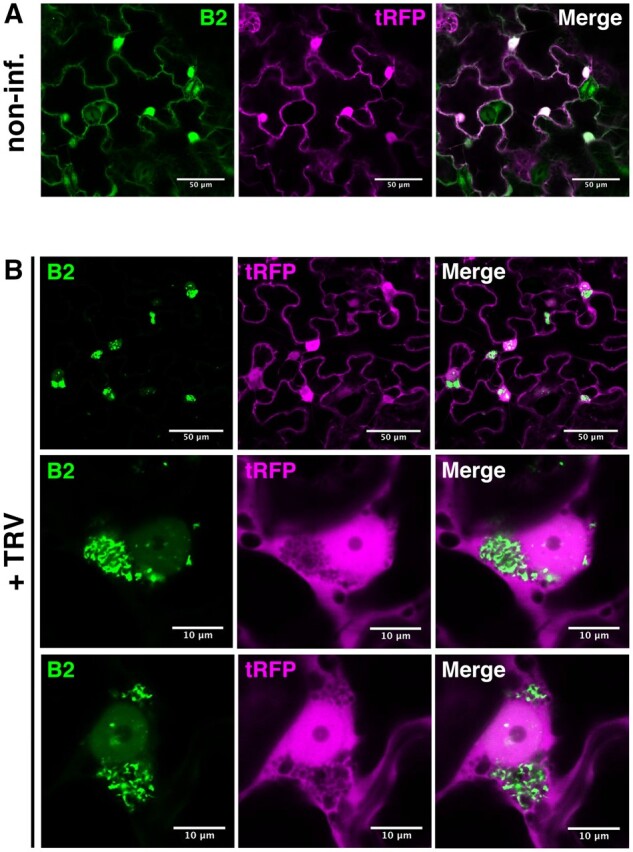
tRFP does not relocalize to TRV replication complexes. Laser confocal microscopy of 35S:*B2:GFP/N. benthamiana* leaves transiently expressing 35S:*tRFP*. A, Acquisition from noninfected leaf disks (×20 objective). Scale bars indicate 50 μm. B, Acquisition of TRV-PDS-infected leaf disks (×20 objective, top), focused on TRV replication complexes (×63 objective, middle and bottom). Scale bars indicate 50 and 10 μm, respectively.

As an absolute prerequisite to our validation pipeline of candidate proteins and considering tRFP was used as reporter tag, we first carefully analyzed the intracellular distribution of tRFP with respect to TRV replication complexes in 35S:*B2:GFP*/*N. benthamiana*. As expected, tRFP as well as B2:GFP showed a typical nucleocytoplasmic localization in cells from healthy plants (Figure 3A), well in agreement with our previous report using the same experimental system (Monsion et al., 2018). Crucially, upon infection the intracellular distribution of tRFP remained unchanged, while B2:GFP concentrated into bright cytoplasmic cotton-ball-like structures often adjacent to the nucleus (Figure 3B). These large structures were previously shown to correspond to TRV viral factories enriched in mitochondria-derived membranes (Monsion et al., 2018) on which replication of TRV is thought to occur (Otulak et al., 2015). Importantly, our data clearly show that while B2:GFP is highly enriched in TRV replication factories, tRFP alone is significantly depleted from these structures (Figure 3B), in agreement with the behavior of tRFP as cytoplasmic and validating tRFP as a reporter protein with which to tag the candidates of interest.

### DRBs perfectly colocalize with B2-labeled viral replication complexes

**Figure 4:**
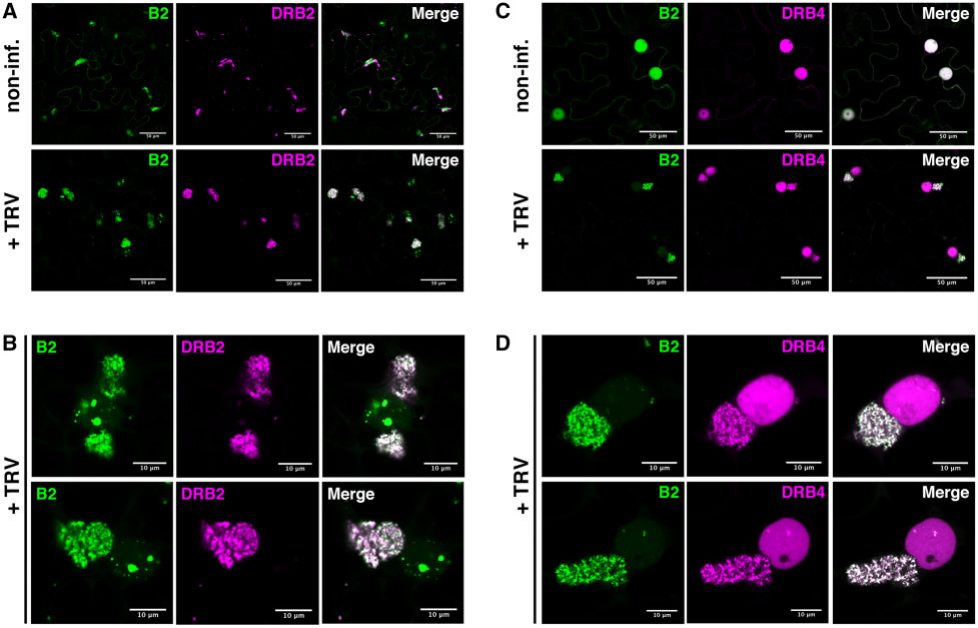
*Arabidopsis thaliana* dsRNA-binding proteins localize at TRV replication complexes. Laser confocal microscopy on 35S:*B2:GFP/N. benthamiana* leaves transiently expressing 35S:*DRB2:tRFP* (A and B) or 35S:*DRB4:tRFP* (C and D). A, Acquisitions with ×20 objective of noninfected (top) and TRV-PDS-infected (bottom) leaf disks expressing DRB2:tRFP. Scale bars indicate 50 μm. B, Acquisitions with ×63 objective of TRV-PDS-infected leaf disks of tissue described in (A). Scale bars indicate 10 μm. C and D, As in (A and B), but from tissue expressing DRB4:tRFP.

DRBs are proteins with dual dsRNA-binding motifs with five representatives in the Arabidopsis genome (Schauer et al., 2002; Clavel et al., 2015). Despite showing low spectral counts in our IPs, two DRBs were identified in our analysis: DRB2 (AT2G28380, total counts: 4, Figure 2A) and DRB4 (AT3G62800, total counts: 5, Figure 2A) that were obvious candidates to test (Figure 4).

DRB2 localizes to the replication complexes of different RNA viruses (Barton et al., 2017), binds dsRNA (Tschopp et al., 2017) and plays a role in endogenous small RNA biogenesis (Pélissier et al., 2011; Eamens et al., 2012; Clavel et al., 2015). In noninfected plants DRB2:tRFP and B2:GFP localized to partially overlapping cytoplasmic and nuclear structures. Interestingly, overexpression of DRB2 changed the localization pattern of B2 from a predominantly nuclear localization (Figure 3A;Monsion et al., 2018) to DRB2-labeled cytoplasmic structures as if B2:GFP was recruited to DRB2 localization sites (Figure 4A). Remarkably, such redistribution of B2 was not observed upon overexpression of DRB4 (Figure 4C). Crucially, near-perfect colocalization of DRB2:tRFP and B2:GFP was observed in the VRCs upon TRV-PDS infection (Figure 4A), which was particularly evident at high magnification (Figure 4B). Moreover, while DRB2:tRFP was almost exclusively found in the cytoplasmic VRCs upon infection, a substantial fraction of B2:GFP remained associated to nuclear structures likely containing dsRNA (Figure 4B). This suggests that although both proteins bind dsRNA, their intracellular targeting is likely not exclusively dsRNA-dependent.

DRB4 has been shown to be both a cofactor of DCL4 in small RNA biogenesis and an inhibitor of DCL3 in endogenous inverted-repeat RNA processing in *A. thaliana* (Fukudome et al., 2011; Montavon et al., 2017). More relevant here, DRB4 is involved in the defense against RNA viruses (Qu et al., 2008; Jakubiec et al., 2012). When we expressed DRB4:tRFP in noninfected tissue, this protein accumulated predominantly to the nucleus where it colocalized with B2:GFP (Figure 4C), in agreement with previous reports (Barton et al., 2017; Monsion et al., 2018). Upon TRV infection DRB4:tRFP was clearly redistributed to VRCs, where it perfectly colocalized with B2:GFP (Figure 4, C and D). In contrast to DRB2 that was barely detectable in the nucleus (Figure 4B), a significant fraction of DRB4:tRFP remained nuclear upon infection (Figure 4, C and D).

Altogether, the robust colocalization of both dsRNA-binding proteins DRB2 and DRB4 with B2:GFP during infection provide (1) further evidence that the TRV-viral factories are indeed cytoplasmic dsRNA hotspots and, more importantly, (2) a validation of the IP procedure.

### Proteins previously linked to viral infection localize at/near VRCs

A family of proteins that emerged with high spectral counts were those belonging to the HSP70 family: HSP70 (AT3G12580, 63 counts); HSP70-1 (or HSC70-1, AT5G02500, 81 counts); and HSP70-3 (or HSC70-3, AT3G09440, 71 counts; Figure 2A). Members of this family of chaperones have been shown in several studies to play key roles in virus infection cycles (reviewed in Verchot, 2012; Nagy, 2016). They can regulate viral life cycles both positively and negatively, and depending on the virus, they affect VRC formation, virus movement, and coat protein homeostasis, among other processes. Three recent studies showed that unrelated plant viruses hijack HSP70 to greatly enhance virus replication (Pogany and Nagy, 2015; Lohmus et al., 2017; Yang et al., 2017).

**Figure 5:**
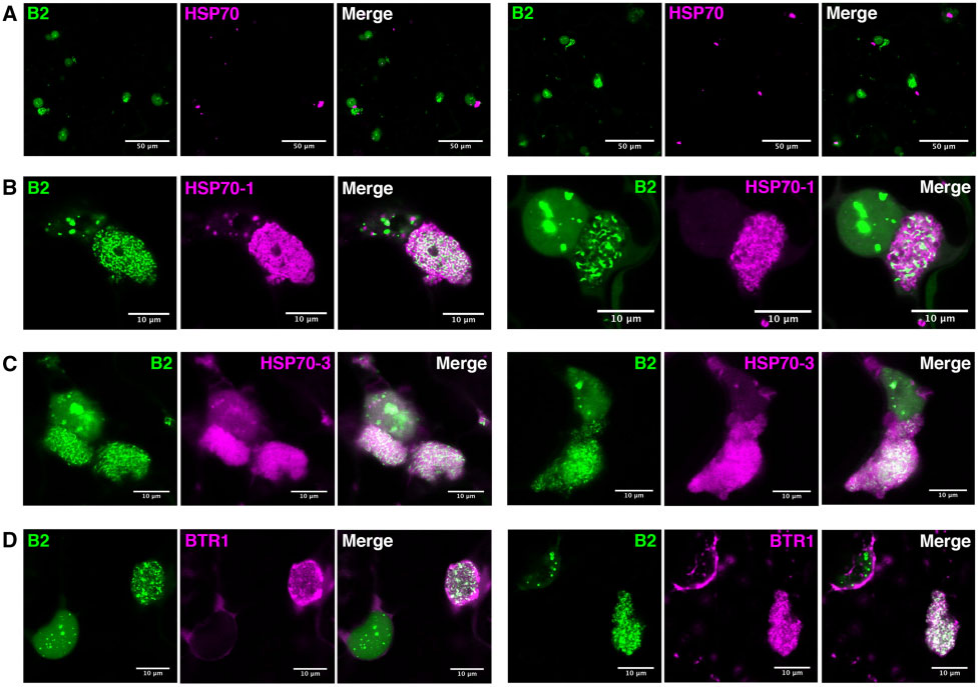
Proteins previously implicated in viral life cycle localize at or near the replication complexes. Laser confocal microscopy on 35S:*B2:GFP/N. benthamiana* TRV-PDS-infected leaf disks transiently expressing (A) 35S:*HSP70:tRFP*, (B) 35S:*HSP70-1:tRFP*, (C) 35S:*HSP70-3:tRFP*, and (D) 35S:*BTR1*:*tRFP*. Acquisitions in (A): ×20 objective, scale bars indicate 50 μm. Acquisitions in (B, C, D): ×63 objective, scale bars indicate 10 μm.

All three HSP70 members were tested in TRV-infected and noninfected 35S:*B2:GFP*/*N. benthamiana* (Figure 5; Supplemental Figure S4). When overexpressed in healthy plants HSP70:tRFP, HSP70-1:tRFP, HSP70-3:tRFP located essentially to distinct cytoplasmic foci whose number, size, and distribution were specific for each of the three HSP70 observed (Supplemental Figure S4). Remarkably, upon infection, HSP70-1:tRFP (Figure 5B) and HSP70-3:tRFP (Figure 5C) were clearly redistributed to TRV viral factories enriched in B2:GFP. In contrast, the localization pattern of HSP70:tRFP remained essentially unaffected upon infection, with no obvious colocalization of B2:GFP with HSP70:tRFP-labeled foci (Figure 5A;Supplemental Figure S4A).

It should be noted that despite the clear redistribution of HSP70-1:tRFP and HSP70-3:tRFP upon infection, only partial colocalization was detected between these proteins and B2:GFP (Figure 5, B and C). The latter appeared engulfed in large HSP70-1 or HSP70-3-containing bodies, likely corresponding to larger viral factories. This sublocalization is in sharp contrast to the near perfect colocalization of B2:GFP with DRB2 and DRB4 upon infection (Figure 4). Altogether our results suggest that HSP70-1 and HSP70-3, contrarily to HSP70, are components of the TRV viral factories. However, in contrast to B2, DRB2 and DRB4, which directly interact with dsRNA, HSP70-1 and HSP70-3 are likely involved in indirect interactions with TRV replication complexes, perhaps via the TRV replicase or other viral or host components. Interestingly, an initial study using the B2:GFP system revealed that dsRNA-containing VRCs constitute only a part of the structures induced by PVX, which in fact also contain viral ssRNA and coat protein (Monsion et al., 2018). The components and activities harbored within these larger “viral factories” are still largely unknown, but the localization patterns of HSP70-1 and HSP70-3 suggest that these proteins associate not only to replication complexes but also to other entities within viral factories.

Next, we tested the localization of an RNA-binding protein present in our IP MS list that was previously shown to associate to plant virus RNA. This protein, known as binding to ToMV RNA (BTR1, AT5G04430, 7 counts; Figure 2A), was identified through affinity purification of tagged viral RNA and found in vitro to bind to the 5′ region of the (+) polarity RNA of ToMV, a *tobamovirus* (Fujisaki and Ishikawa, 2008). In our experimental system, BTR1:tRFP localized to numerous cytoplasmic punctate structures at the cell periphery in noninfected cells (Supplemental Figure S4D). Upon TRV-PDS infection, BTR1 relocalized mainly to B2:GFP-labeled VRCs (Figure 5D), while a fraction was maintained at sites similar to those seen in noninfected cells (Supplemental Figure S4D). At high magnification it is possible to see that BTR1 did not strictly and exclusively colocalize with B2:GFP, but could also be seen in the areas surrounding B2:GFP-labeled dsRNA hotspots (Figure 5D). Similar to HSP70-1 and HSP70-3, it is possible that BTR1 associates not only to VRCs but also to other entities within viral factories.

### Proteins not previously linked to infection localize at/near replication complexes

Among the potential TRV replication complex-associated proteins identified through IP, we tested three for which we found no specific function in virus process from the literature: a RING/U-box protein (AT3G45570, 50 counts) and Ribosomal Protein S27a (AT1G23410, 82 counts) and NFD2 (Nuclear Fusion Defective 2—AT1G24450, 12 counts; Figure 2A).

The RING/U-box protein, which we will refer to as RUP1, belongs to the E3 ubiquitin ligase RBR family. The N-terminal half of the protein is homologous to the RNAse H superfamily, followed on the C-terminal half by a RING-type zinc-finger domain and an IBR (in between ring fingers) domain. The Ribosomal Protein 27a, here abbreviated as RP27a, is a small protein of 156 amino acids with a ubiquitin domain N-terminal half and a zinc-binding ribosomal protein superfamily C-terminal section. Importantly, all RP27a peptides detected in B2:GFP coimmunoprecipitated samples belong to the ubiquitin domain, and also match with the protein sequences of UBQ1 through UBQ14 (Supplemental Figure S5). This suggests that one or more proteins present in the immunoprecipitates and possibly associated to TRV replication complexes are ubiquitinated.

To investigate which protein/s were ubiquitinated, we searched the MS dataset for diglycine footprints, a hallmark of ubiquitination. Interestingly, the only protein found to contain such a feature was RP27a itself, only on lysine-48 (Supplemental Figure S5), suggesting self-ubiquitination and/or the formation of lysine-48 polyubiquitin chains. Given that no other diglycine footprint was found in our spectrometry dataset, the proteins targeted by these chains may have been below detection level and remain to be identified. Finally, NFD2 was first identified as a factor involved in karyogamy, the fusion of polar nuclei within the central cell of the female gametophyte prior to fecundation and the fusion of the sperm cells’ nuclei with the egg cell and the central cell upon fecundation (Portereiko et al., 2006). This protein, containing an RNAse III domain, has been also described as RNASE THREE-LIKE 4 (RTL4; Elvira-Matelot et al., 2016).

**Figure 6:**
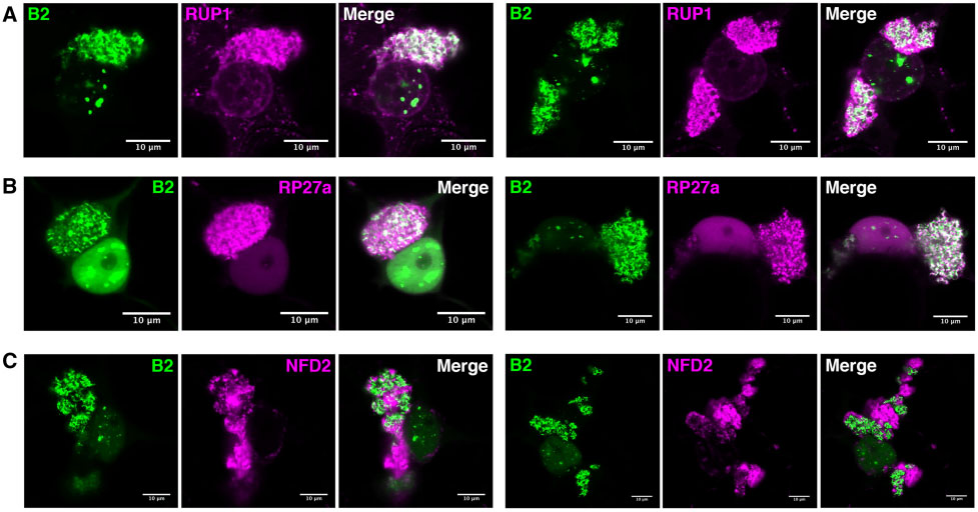
Localization of previously undescribed proteins at or near the replication complexes. Laser confocal microscopy (×63 objective) on 35S:*B2:GFP/N. benthamiana* TRV-PDS-infected leaf disks transiently expressing (A) 35S:*RUP1*:*tRFP*, (B) 35S:*tRFP:RP27a*, and (C) 35S:*tRFP:NFD2*. Scale bars indicate 10 μm.

In healthy cells, tRFP-tagged RUP1 and RP27a displayed a nucleocytoplamic distribution (Supplemental Figure S6, A and B), while NFD2 was essentially found in numerous cytoplasmic bodies (Supplemental Figure S6C). Upon TRV infection, all three proteins were clearly relocalized to or near B2:GFP-labeled dsRNA hotspots (Figure 6; Supplemental Figure S6). More precisely, RUP1 and RP27a showed patterns similar to those observed with HSP70-1, with extensive overlap with B2:GFP as seen from the white color in the merged panels (Figure 6, A and B). This suggests that RUP1 and RP27a associate not only with VRCs but also to other entities within viral factories.

Interestingly, NFD2 showed a pattern of localization different from the other proteins tested in this work (Figure 6C;Supplemental Figure S6C). Although localization of NFD2 and B2 seemed mutually exclusive, NFD2 being absent from B2:GFP-labeled structures and vice versa, a continuum between B2:GFP-labeled hotspots and NFD2-labeled structures was observed (Figure 6C), suggesting that NFD2 is intimately linked to TRV-induced subcellular entities and was therefore immunoprecipitated. In addition, the complete localization of NFD2 in close proximity to TRV replication complexes was in stark contrast with the perinuclear and cytoplasmic point-form localization of NFD2 in noninfected plants **(**Supplemental Figure S6C).

### Knockout of DRB2 potentiates TRV systemic infection in a Dicer-independent manner

Next, we tested whether genetic knockout of the candidate genes analyzed would lead to changes in TRV systemic accumulation. To do so, we acquired *A. thaliana* lines with T-DNA insertions in the genes of interest: *drb2-1*, *drb4-1*, and *drb2-1 drb4-1* (Curtin et al., 2008), *btr1-1* (Fujisaki and Ishikawa, 2008), SALK_078851 (*rup1*), SALK_093933 (*rp27a*), SALK_088253 (*hsp70*), SALK_135531 (*hsp70-1*), and SAIL_178_E10 (*hsp70-3*; Supplemental Figure S7). No lines carrying insertions in the annotated 5′UTR or coding sequence of *NFD2* were found.

**Figure 7:**
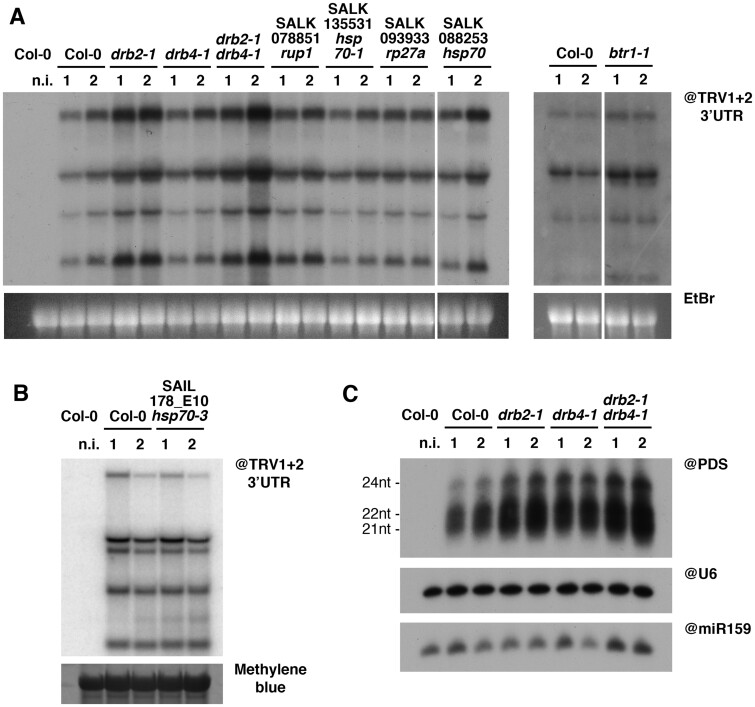
Knockout of DRB2 causes increased systemic accumulation of TRV in Arabidopsis, through a mechanism independent from small RNA biogenesis. A and B, RNA gel blot analysis of RNA from systemically infected leaves of Arabidopsis knockout lines infected with TRV-PDS, 12 dpi. Previously published mutants are indicated with their current name, while the others are indicated with their SALK/SAIL nomenclature. Each sample is a pool of four to five plants, and two samples were analyzed per genotype (1 and 2), per time point. EtBr (A) or methylene blue (B) staining were used as loading control. C, PAGE RNA gel blot analysis of small RNA from the corresponding samples in (B). snU6 and miR159 were used as loading controls.

We infected 10 plants per genotype and harvested the systemically infected leaves 12 days postinfection (dpi), dividing the plants of each genotype into two equal pools. RNA gel blot analysis of the total RNA from these samples revealed that both the single *drb2-1* and double *drb2-1 drb4-1* mutants showed markedly increased TRV accumulation in systemic leaves compared to control Col-0 plants (Figure 7, A and B). A parallel experiment on inoculated leaves 3 dpi showed that none of the mutants tested affected TRV local accumulation (Supplemental Figure S8). A further experiment confirmed increased TRV accumulation in systemic leaves of *drb2-1* (Supplemental Figure S9) and in an independent T-DNA mutant, *drb2-2* (Sawano et al., 2017). The *drb2-2* mutant showed a milder increase in TRV accumulation compared to the *drb2-1* mutant, which we attribute to the fact that in *drb2-2* the T-DNA insertion is at the 3′ proximal end of the *DRB2* ORF. This likely results only in a partial knock out of the DRB2 protein function, as opposed to the *drb2-1* T-DNA insertion at the 5′ end of the gene. These results combined suggest that DRB2 could play an antiviral function with respect to systemic infection by TRV. A moderate increase in TRV accumulation was also observed in systemic leaves of *btr1-1* mutants (Figure 7A). Similar effect on ToMV accumulation was reported by Fujisaki and Ishikawa (2008).

Considering that major differences in TRV accumulation between mutant lines and Col-0 control were essentially restricted to *drb2-1* and *drb2-1 drb4-1* lines, we decided to focus our attention on possible antiviral function of DRB2. Since DRB proteins have been shown by several studies to be involved in small RNA biogenesis (Fukudome et al., 2011; Pélissier et al., 2011; Jakubiec et al., 2012; Montavon et al., 2017; Tschopp et al., 2017) and *DRB2* genetic knockout has been shown to impact accumulation of several microRNAs (Eamens et al., 2012), we decided to analyze the viral siRNA (vsiRNA) present in the *drb* mutants described above (Figure 7C). RNA gel blot analysis of small RNAs revealed an increase in vsiRNA in the *drb2-1 and drb2-1 drb4-1* mutants analyzed. This most likely reflects the increase in TRV genomic RNAs in these samples (Figure 7A), which are substrates for vsiRNA biogenesis. Moreover, DRB2 knockout did not cause any noticeable changes in the vsiRNA size distributions. These observations, overall, lead us to conclude that knockout of DRB2 (1) positively impacts TRV RNA and vsiRNA steady-state levels and (2) does not cause changes in the respective contributions of DCL2, DCL3, and DCL4 to this process. These observations are in line with what has been previously observed for TuMV and TSWV (Curtin et al., 2008). Therefore, the increase of TRV systemic accumulation observed in *drb2* mutants is likely not due to impaired dicing activity, a step upstream in the RNA silencing pathway that is normally associated to DRB proteins.

### DRB2 overexpression drastically reduces the accumulation of various plant RNA viruses

**Figure 8:**
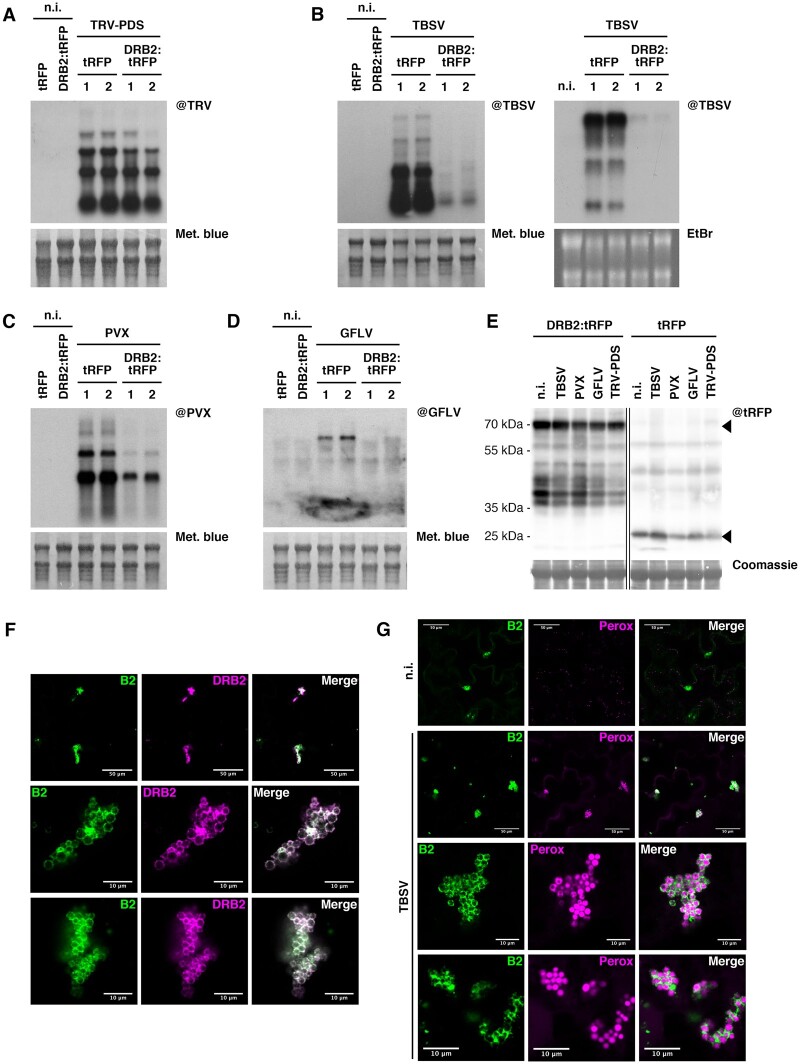
Overexpression of DRB2 in *N. benthamiana* leaves drastically reduces accumulation of a wide range of RNA viruses. A, Northern blot analysis of RNA from *N. benthamiana* leaf disks 4 days after transient transformation with *35S:tRFP* or *35S:DRB2:tRFP* and 3 dpi with TRV-PDS (except for n.i.). Each sample is a pool of 40–50 leaf disks from 4–5 leaves. In the case of the virus-infected leaves, two samples were analyzed per condition (indicated with 1 and 2). Methylene blue staining of the membrane was used as loading control. B, As in (A), but after rub-inoculation with TBSV. Two independent biological replicates are shown with either methylene blue or EtBr staining of the membranes as loading control. C, As in (A), but after rub-inoculation with PVX. D, As in (A), but after rub-inoculation with GFLV. E, Immunoblot analysis on protein extracts from the samples analyzed in (A–D), to detect DRB2:tRFP (top arrowhead) and tRFP (bottom arrowhead). Coomassie blue staining was used as loading control. Source data are available with the Blotting Source Data. F, Laser confocal microscopy acquisitions of B2-labeled TBSV replication complexes, from 35S:*B2:GFP*/*N. benthamiana* plants transiently expressing DRB2:tRFP and infected with TBSV. Scale bars indicate 50 (top) or 10 μm (middle and bottom). G, As in (F), but from plants (noninfected in the top row, TBSV-infected in the rest) transiently expressing the peroxisome marker tRFP-SKL. Scale bars indicate 50 (top two acquisitions) or 10 μm (bottom two acquisitions).

The absence of DRB2 resulting in increased TRV accumulation (Figure 7), we next tested whether AtDRB2 overexpression could negatively impact infection by TRV and possibly by other distantly related RNA viruses. To this end, agroinfiltrated *N. benthamiana* leaves transiently expressing DRB2:tRFP or tRFP were mechanically inoculated with the viruses of interest, and 3 days after infection leaf disks were collected. RNA gel blot analysis revealed that in tissues infected by TRV-PDS (Figure 8A), TBSV (Figure 8B), *Potato virus X* (PVX; Figure 8C), and *Grapevine fanleaf virus* (GFLV; Figure 8D), overexpression of DRB2:tRFP leads to a dramatic decrease in virus accumulation compared to over-expression of tRFP alone. This effect was particularly prominent for TBSV, PVX, and GFLV, despite the presence of comparable amounts of DRB2:tRFP (Figure 8E).

Remarkably, confocal microscopy of B2:GFP-expressing *N. benthamiana* leaves transiently overexpressing DRB2:tRFP and infected with TBSV showed that DRB2:tRFP colocalizes with VRCs (Figure 8F) that are structurally different from those produced upon TRV infection (Figure 4B**)**. To confirm that these are indeed TBSV VRCs, which are known to form on peroxisome membranes (Nagy et al., 2016), we generated a clone to express a tRFP-SKL peroxisome marker (Incarbone et al., 2018). Expression of this marker in B2:GFP-expressing *N. benthamiana* leaves subsequently infected with TBSV reveal that B2-labeled VRCs are indeed localized on the surface of peroxisomes, that in infected conditions appear to group into large peroxisome clusters (Figure 8G). These results clearly show that *At*DRB2 localizes to VRCs from different viruses and is a broad-ranged and potent antiviral effector.

## Discussion

Here, we describe an approach for the identification of VRC-associated proteins through the isolation of replicating viral dsRNA during genuine infection, and validated the localization of most of the candidates through a rapid, robust, and simple system. We also showed that one of the proteins we identified as associated to viral dsRNA, DRB2, has antiviral activity against several RNA viruses that belong to different taxonomic groups (Koonin et al., 2020): GFLV (phylum: *Pisoviricota*, class: *Pisoniviricetes*, order: *Picornavirales*, family: *Secoviridae*), TRV (phylum: *Kitrinoviricota*, class: *Alsuviricetes*, order: *Martellivirales*, family: *Virgaviridae*), TBSV (phylum: *Kitrinoviricota*, class: *Tolucaviricetes*, order: *Tolivirales*, family: *Tombusviridae*), and PVX (phylum: *Kitrinoviricota*, class: *Alsuviricetes*, order: *Tymovirales*, family: *Alphaflexiviridae*). Although the proof of concept for our approach to identify VRC-associated proteins is established here only for TRV, it should be compatible with any plant virus as long as it is able to produce dsRNA during its replication cycle. Importantly, it does not involve as a prerequisite any modification of viral genomes, the production of infectious clones or the specific tagging of viral protein. Also, considering that the isolation of viral dsRNA and associated proteins is achieved indirectly by anti-GFP antibodies, there is no requirement for virus- or dsRNA-specific antibodies in the process. Hopefully, this experimental approach will provide future investigators with a universal tool to successfully explore the proteome associated to the replication complexes of their favorite RNA virus, which can then be studied more in detail to discover the function of VRC-associated proteins and their involvement in the viral life cycle. As hosts, 35S:*B2:GFP*/*A. thaliana* (this study) and 35S:*B2:GFP*/*N. benthamiana* (Monsion et al., 2018) are compatible with numerous plant virus species. If needed, the systems could be easily adapted to other plant species, as long as they accommodate stable transformation.

We have shown that ectopic expression of B2:GFP greatly increases the accumulation of TRV RNA both in *A. thaliana and N. benthamiana*. Given the activity of the 73 amino-acid double-stranded binding domain of B2 as a VSR (this work), it is tempting to ascribe TRV overaccumulation simply as a consequence of RNA silencing suppression and subsequent enhanced viral replication. While this is probably the case, it cannot be excluded that B2:GFP increases TRV accumulation by RNAi-independent means, such as stabilization of dsRNA or its protection from other host defensive pathways (Li and Wang, 2019).

The drastic effect of B2:GFP on TRV infection can be viewed as a double-edged sword in relation to its use as bait to pull down VRCs. On the one hand, this effect may introduce biases of both quantitative and qualitative nature, such as the unspecific association to VRCs of host proteins that do not play a role during infection in wild-type conditions or changes in the accessibility or protein complement of replicating RNA, for example. On the other hand, the overaccumulation of TRV dsRNA constitutes a real advantage for the study of VRCs. In fact, increased viral replication is in favor of (1) a better IP efficiency; (2) an enhanced detection of protein partners by MS; and (3) an improved visualization of VRCs with test candidates. While these biases clearly need to be taken into account, we strongly believe that overall, this approach has far more advantages than drawbacks.

The abundance of TRV replicase detected in the IPs (624 reads; Figure 2A) is, in our opinion, confirmation of the robustness of the experiment in terms of VRC yield and integrity. The abundant detection of the coat protein suggests that it either plays a direct role in TRV replication or that the VRCs present in the IPs contain not only full-length dsRNA, but also (+)ssRNA that is being encapsidated, possibly during or just after separation from the (−) strand. However, despite the use of detergent during the IPs, it is possible that we have pulled down proteins present on membranes or complexes close to the replication organelles but not actually part of them. Furthermore, B2 can potentially bind dsRNA generated by secondary structures in viral ssRNA as well as dsRNA produced by host RDR proteins. Future work involving B2 IPs combined with RNA sequencing in WT Arabidopsis as well as mutants for RDR genes, will shed further light on the issue.

Remarkably, among the nine candidate *A. thaliana* proteins detected following B2 IP and tested in this work, only one, HSP70, failed to accumulate in VRCs despite a high spectral count. At this stage, one cannot strictly rule out the possibility that HSP70, in contrast to HSP70-1 and HSP70-3, corresponds to a false positive despite being enriched in the IPs. It is also possible that tRFP could have disrupted the function of the protein or that the *A. thaliana* HSP70 (AT3G12580) may not be fully functional when expressed in the heterologous host *N. benthamiana.* It should be noted however that HSP70 has been linked to viral infection in a number of studies (Verchot, 2012; Nagy, 2016) and found to directly bind the viral replicase of at least two viruses (Nishikiori et al., 2006; Serva and Nagy, 2006). Also, Arabidopsis mRNA-interacting proteome includes members of the Hsc70/Hsp70 family (Reichel et al., 2016) well in agreement with the RNA-binding functions of the human Hsp70 that was shown to be independent of the protein chaperone activity (Kishor et al., 2017). This raises the possibility that HSP70 family members may associate with viral factories via RNA binding.

All remaining eight candidates were specifically redistributed upon infection, suggesting involvement of these factors in the viral life cycle. Their localization patterns can be divided into three groups: perfect colocalization (DRB2 and DRB4), partial colocalization (HSP70-1, HSP70-3, BTR1, RUP1, and RP27a), and proximity (NFD2). Perfect colocalization most likely reflects the direct association of DRB2 and DRB4 on replicating dsRNA within the VRCs. This result is in line with the experimentally verified ability of DRB2 and DRB4 to bind dsRNA (Kobayashi et al., 2009; Tschopp et al., 2017) and of DRB4 to bind TYMV dsRNA in vivo (Jakubiec et al., 2012). DRB2 in *A. thaliana* relocalizes to cytoplasmic punctate bodies upon infection by TuMV, TSWV, and TYMV (Barton et al., 2017) and DRB4 relocalizes from nuclei to cytoplasmic VRCs upon TYMV infection (Jakubiec et al., 2012). While DRB4 plays a role in antiviral defense (Qu et al., 2008; Jakubiec et al., 2012) as part of the RNA silencing machinery, the function of DRB2 recruitment to replication complexes remains to be uncovered.

Although additional experiments are required to confirm the direct association of DRB2 and DRB4 to TRV replicating dsRNA and DRB2 to TBSV replicating dsRNA, our data suggest that host proteins including antiviral defense protein such as DRBs may have access to viral dsRNA within replication organelles including TBSV-induced spherules. This potentially questions the suggested function (or at least efficiency) of replication organelles as protective structures against degradation by cellular RNases and detection by putative dsRNA sensors that trigger antiviral responses (Romero-Brey and Bartenschlager, 2014; Nagy et al., 2016; Fernandez de Castro et al., 2017). It is conceivable that B2:GFP and the DRB proteins gain access to viral dsRNA at early stage of replication organelle morphogenesis before replication complexes become eventually fully protected.

While the precise molecular pathways linking DRB2 to VRCs remain to be uncovered, we have shown through genetic ablation and overexpression that this protein is a key element in the host’s restriction of viral systemic infection. We have also shown that the antiviral activity of DRB2 likely does not involve Dicer function, since vsiRNA production remains unchanged upon knockout of DRB2. Our data, however, do not rule out a possible involvement of DRB2 in steps of the RNA interference pathway that are downstream of Dicer processing. Whatever the molecular mode of action of Arabidopsis DRB2, our overexpression experiments have shown that heightened production of this protein in planta drastically reduces the accumulation of viruses belonging to various families. In contrast with our observations, a recent study has shown that the DRB2B protein of *N. benthamiana*, when overexpressed, strongly increased PVX accumulation (Fatyol et al., 2020). While in this study no other virus species were tested, these differences between *A. thaliana* DRB2 and *N. benthamiana* DRB2B suggest that the functions of these gene products in relation to RNA viruses are likely not conserved. However, it is also possible that another of the DRB2 homeologs present in the allotetraploid *N. benthamiana* genome, and not tested in the aforementioned study, could have the antiviral effect observed for *A. thaliana* DRB2. In light of our results, we believe that further study of Arabidopsis DRB2 and its use as a biotech tool in crop defense against viral infection hold substantial potential.

The pattern of partial colocalization, observed for HSP70-1, HSP70-3, BTR1, RUP1, and RP27a/Ubiquitin, consisted in the localization at the B2-labeled VRCs per se, as well as features in close proximity, generally designated as “viral factories.” In the case of PVX, the dsRNA-containing replication complexes reside within larger viral factories harboring other viral proteins and viral ssRNA (Monsion et al., 2018). In general, these viral factories are most likely the hub for a plethora of viral activities beyond RNA replication *sensu stricto*, such as translation, encapsidation, etc., and which likely require specific host-encoded proteins. Our work suggests that HSP70-1, HSP70-3, BTR1, RUP1, and RP27a/Ubiquitin may play such functions during replication of TRV and possibly other viruses. Indeed, HSP70-3 and BTR1 have been shown to interact with TuMV replicase (Dufresne et al., 2008) and ToMV ssRNA (Fujisaki and Ishikawa, 2008), respectively. Similarly, ubiquitin and the ubiquitin pathway have been shown in a number of studies to play important roles in plant virus life cycle, both pro-viral and anti-viral, the details of which are exhaustively reviewed in (Alcaide-Loridan and Jupin, 2012; Verchot, 2016). Concerning NFD2, the pattern of proximity suggests that this protein may indirectly be involved in viral factory function without direct association with viral dsRNA per se. The fact that genetic knockout of most of these factors did not lead to drastic changes in viral RNA accumulation (with the notable exception of DRB2) does not rule out their involvement in viral functions despite their localization to VRCs. They could act redundantly with other proteins, or could affect parameters that do not perturb viral RNA accumulation, to name but a few possibilities. The genetic dissection of the roles played by the proteins here identified, through experiments including IP and MS of tagged alleles of these factors in different genetic backgrounds, is outside the scope of this manuscript and will be addressed in further studies.

Finally, we have previously shown that B2:RFP can be used to mark *Drosophila C virus* replication complexes in insect cells in vivo (Monsion et al., 2018), suggesting that B2:GFP could be used as a tool to pull down replication complexes of RNA viruses in organisms other than plants. Since dsRNA is a key replication intermediate of all RNA viruses, we believe the further development and adaptation of this tool also holds great potential for the investigation of RNA viruses infecting humans.

## Materials and methods

### Plant material and growth conditions

Transgenic 35S:*B2:GFP*/*Nicotiana benthamiana* plants were previously described (Monsion et al., 2018). Transgenic *A.* *thaliana* plants (Col-0 line and genetic backgrounds including mutants of the core antiviral Dicer-Like genes, *dcl2-1*, *dcl4-2*, and triple *dcl2-1 dcl3-1 dcl4-2* lines (Deleris et al., 2006) expressing 35S:*B2:GFP* were generated using same plasmid (pEAQΔP19-B2:GFP) and agrobacteria described in (Monsion et al., 2018), following floral dip transformation (Harrison et al., 2006) with addition of Plant Preservative Mixture (Plant Cell Technology) at 2 ml L^−1^ in Murashige and Skoog medium. Individual Arabidopsis transformed lines were self-pollinated to generate (F3) plants homozygous for the transgene. T-DNA insertion in the genes of interest were confirmed in the SALK/SAIL mutant lines through standard PCR-based genotyping, using primers listed in Supplemental Table S1. Refer to Supplemental Figure S7 for T-DNA insertion sites and results of PCR-based genotyping.

*Nicotiana* *benthamiana* plants were grown in a greenhouse at 22°C–18°C, 16h/8h light/dark photoperiod, while *A. thaliana* were grown in a neon-lit growth chamber at 22°C–18°C, 12h/12h light/dark photoperiod.

### Golden Gate pEAQΔP19 vector construction

Binary vector pEAQΔP19-GG was obtained by (1) removing 3 SapI restriction sites present in pEAQ-*HT* (Sainsbury et al., 2009); (2) inserting a Golden Gate cassette (similar to Gateway without AttR1/2) with SapI sites at extremities; and (3) removing P19. Two silent substitutions into Neomycin phosphotransferase (nptII) gene and one substitution near the origin of replication (ColE1) were produced by PCR mutagenesis using Phusion polymerase in GC buffer supplemented with 5% DMSO (primers in Supplemental Table S1, nos. 595−596 and 638−641) in order to obtain plasmid pEAQ-HT-ΔSapI. A Golden Gate cassette amplicon (pEAQ-HT as matrix, see primer no. 589 + 642; Supplemental Table S1) was inserted via AgeI/XhoI restriction sites in pEAQ-HT-ΔSapI. Finally, P19 was excised by double restriction EcoNI/SgsI (FD1304, FD1894, Thermo Scientific), extremities were filled in with Klenow fragment (EP0051, Thermo Scientific), supplemented with dNTPs, followed by a ligation step and transformation in *Escherichia* *coli* (ccdB Survival strain, Invitrogen).

### Cloning of candidate genes

Candidate genes were amplified from *A. thaliana* genomic DNA with primers designed to contain SapI restriction sites compatible with Golden Gate cloning (Engler et al., 2009) and adapters necessary for ligation to an N-terminal or C-terminal tag (primer list in Supplemental Table S1). In the case of genes containing SapI restriction sites, silent mutations were introduced to remove these sites through overlap PCR. In parallel, tRFP was amplified with primers designed to contain SapI restriction sites, adapters for ligation to the N- or C-terminal end of the candidate gene, and a peptide linker (GGGSGGG amino acid sequence) between tRFP and the candidate gene. tRFP-SKL was generated by adding the bases to encode the SKL tripeptide in the reverse primer before the stop codon. PCR products were purified from agarose gel and used in a Golden Gate reaction containing the candidate gene, tRFP, binary vector pEAQΔP19-GG, SapI (R0569L, New England Biolabs), CutSmart buffer (New England Biolabs), T4 DNA ligase 5 Units µL^−1^ (EL0011, Thermo Scientific), and 0.5 or 1 mM ATP (R0441, Thermo Scientific). Golden Gate reaction cycling: 10 cycles of 37°C 10 min, 18°C 10 min; 18°C 50 min, 50°C 10 min, 80°C 10 min. Following transformation in *E. coli* (TOP10 strain, Invitrogen), purification and sequencing, plasmids were transformed into *A. tumefaciens* strain GV3101. pEAQΔP19-B2:RFP served as matrix to generate plasmid pEAQΔP19-B2mut:RFP as described in Monsion et al. (2018) (primers no. 631–632; Supplemental Table S1).

### Plant inoculation, infection, and sampling

For fluorescence microscopy experiments, leaves of 5- to 6-week-old 35S:*B2:GFP/N. benthamiana* were infiltrated with *A. tumefaciens* GV3101 carrying plasmid pEAQΔP19 containing the tagged gene of interest, at absorbance_600__nm_ (A_600_) of 0.2. Prior to inoculation, bacteria were incubated in 10 mM MES pH 5.6, 10 mM MgCl_2_, 200 µM acetosyringone for 1 h. TRV infection was initiated upon agroinfection with bacteria carrying plasmids expressing the two viral genomic RNAs (Liu et al., 2002), at A_600_ 0.01 each. This method of virus delivery was chosen because it results in homogenous and ubiquitous infection throughout the inoculated tissue. About 3–4 days postinoculation, leaf disks of 5 mm in diameter were collected, vacuum-infiltrated with water and the abaxial side of the disks observed by confocal microscopy (see below). *Arabidopsis* *thaliana* infection for B2:GFP IP experiments was carried out as for *N. benthamiana*, with the difference that *A. tumefaciens* was induced by incubating 5–6 h in induction medium (10.5 g L^−1^ K_2_HPO_4_, 4.5 g L^−1^ KH_2_PO_4_, 1 g L^−1^ (NH_4_)_2_SO_4_, 0.5 g L^−1^ sodium citrate, 0.1 g L^−1^ MgSO_4_, 0.4% glycerol, 0.1 g L^−1^ MES, 200 µM acetosyringone), and bacteria used at A_600_ 0.5 each.

For evaluation of TRV local and systemic accumulation in Arabidopsis mutants, sap rub inoculation was used. Briefly, Col-0 leaves systemically infected with TRV-PDS were ground in liquid N_2_, homogenized in 50 mM sodium phosphate buffer pH 7.5, cleared by centrifugation for 2 min at 2,000*g*, transferred to a new tube and kept on ice until inoculation. This was carried out by sprinkling celite on four to five leaves per plant, dipping a cue tip in the inoculum and gently rubbing it onto the leaves. These were then rinsed with water. Systemically infected leaves (the upper seven to eight leaves of each plant) were harvested 12–13 dpi. Samples used for IP experiments consisted of pools of at least 10 plants, while samples for assessment of TRV accumulation consisted of pools of four to five plants each. Sampled leaves were immediately frozen in liquid N_2_ and thoroughly ground to a powder in a mortar to ensure homogenous representation in the subsequent analyses.

For the experiments shown in Figure 8, virus infection was carried out by rub inoculation: the day following agroinfiltration with 35S:*tRFP*, 35S:*tRFP-SKL*, or 35S:*DRB2:tRFP*, the abaxial side of the infiltrated leaves was mechanically inoculated. The inoculum was obtained by grinding frozen *N. benthamiana* tissues infected with TBSV, TRV-PDS, PVX-GFP, or GFLV in 50 mM sodium phosphate buffered at pH 7 (except for TBSV, at pH 5.8). For confocal microscopy, samples were prepared as described above. For virus accumulation assessment, a total of 50–60 leaf disks of 5 mm were taken from inoculated leaves (three plants/condition, four leaves/plant), frozen, and pulverized.

### Immunoprecipitation

IPs were performed as previously described (Incarbone et al., 2017), with minor modifications. 0.15 g of young rosette leaves were ground in liquid nitrogen, homogenized in a chilled mortar with 1 mL lysis buffer (50 mM Tris–HCl, pH 8, 50 mM NaCl, 1% Triton X-100) containing 1 tablet 50 mL^−1^ of protease inhibitor cocktail (Roche), transferred to a tube and incubated for 15 min at 4°C on a wheel. Cell debris was removed by two successive centrifugations at 12,000*g* for 10 min at 4°C, after which an aliquot of supernatant was taken as input fraction. The remaining extract was incubated with magnetic microbeads coated with monoclonal anti-GFP antibodies (μMACS purification system, Miltenyi Biotech, catalog number #130-091-125) at 4°C for 20 min. Sample was then passed through M column (MACS purification system, Miltenyi Biotech) and an aliquot of the flow-through fraction was taken. The M column was then washed two times with 500 μL of lysis buffer and one time with 100 μL of washing buffer (20 mM Tris–HCl, pH 7.5). The beads and associated immune complexes were recovered by removing the M column from the magnetic stand and passing 1 mL Tri Reagent (for subsequent RNA analysis—see dedicated section) or 200 μL hot 1X Laemmli buffer (for protein analysis—see dedicated section). A 4X Laemmli buffer was added to input and flow-through fractions before protein denaturation for 5 min at 95°C.

### RNA extraction and analysis

RNA from total and immunoprecipitated fractions was performed with Tri-Reagent (Sigma) according to manufacturer’s instructions. Briefly, 0.2 g tissue were ground in liquid nitrogen and homogenized in 1 mL Tri-Reagent, 400 μL of chloroform were added, and sample was thoroughly shaken for 2 min. After 10 min spin at 15,000*g*, 4°C, supernatant was added to at least 1 vol isopropanol (and 1.5 μL glycogen in the case of immunoprecipitated samples—IP) and incubated 1 h on ice (O/N for IP). After 15 min spin at 15,000*g*, 4°C (30 min for IP), pellet was washed in 80% ethanol, dried and resuspended in water. RNA was analyzed by RNA gel blot (denaturing agarose gel to detect high molecular weight RNA, denaturing PAGE to detect low molecular weight RNA) and dsRNA–protein blot (native agarose gel to detect long dsRNA). In the RNA gel blot, miRNAs were detected through DNA oligonucleotides labeled with γ-^32^P-ATP using T4 PNK (see Supplemental Table S1). TRV genomic and subgenomic RNAs were detected in the same way, with an oligonucleotide complementary to a part of the 3′UTR sequence common to RNA1 and RNA2. The same was done for TBSV. TRV-PDS-derived siRNAs were detected through PCR-amplified *A. thaliana* PDS sequence labeled by random priming reactions in the presence of α-^32^P-dCTP. The same was done to detect PVX and GFLV RNA in the RNA gel blot. In the dsRNA-protein gel blot, dsRNA were detected with recombinant Strep-Tagged FHV B2, as previously described (Monsion et al., 2018).

Quantitative RT-PCR was performed in the following manner: RNA samples were treated with DNAse (Thermofisher TURBO DNA-free kit) and cDNA was generated with random primers (Thermofisher RevertAid First Strand cDNA synthesis kit), according to manufacturer’s instructions. qPCR was then performed with Roche FastStart Essential DNA Green Master kit on a LightCycler 96, in technical triplicates for each sample and primer combination. Data were analyzed with standard ΔΔCt normalization.

### Protein extraction and analysis

Proteins from total fractions were extracted as previously described (Hurkman and Tanaka, 1986). Immunoprecipitated proteins for MS analysis were isolated as described above, then denatured 5 min at 95°C. Immunoprecipitated proteins from RNA IP were obtained by collecting the phenolic phase following Tri-reagent/chloroform extraction, adding 3–4 vol acetone and incubating at −20°C O/N. After centrifugation (15,000*g*, 15 min, 4°C) pellet was washed in 80% acetone and resuspended in 1X Laemmli. Proteins were resolved by SDS-PAGE and electro-blotted onto Immobilion-P membrane. This was incubated with the appropriate antibodies (anti-GFP polyclonal antibody and anti-tRFP antibody, Evrogen, reference # AB233) and revealed with Roche LumiLight ECL kit after incubation with secondary antibody.

### MS analysis and data processing

Proteins were digested with sequencing-grade trypsin (Promega) and analyzed by nanoflow liquid chromatography (nanoLC) coupled to tandem mass spectrometry (MS/MS) on a TripleTOF 5600 mass spectrometer (Sciex, USA) as described previously (Chicher et al., 2015). Data were searched against the TAIR v.10 database with a decoy strategy (27281 protein forward sequences). Peptides were identified with Mascot algorithm (version 2.5, Matrix Science, London, UK) and data were further imported into Proline v1.4 software (http://proline.profiproteomics.fr/). Proteins were validated on Mascot pretty rank equal to 1, and 1% FDR on both peptide spectrum matches (PSM score) and protein sets (Protein Set score). The total number of MS/MS fragmentation spectra was used to quantify each protein from at least three independent biological replicates. A statistical analysis based on spectral counts was performed using a homemade R package as described in (Lange et al., 2019). The R package uses a negative binomial GLM model based on EdgeR (Robinson et al., 2010) and calculates, for each identified protein, a fold-change, a *P*-value and an adjusted *P*-value corrected using the Benjamini–Hochberg method.

### Confocal laser scanning microscopy

Observations of leaf disks were carried out using Zeiss LSM700 and LSM780 laser scanning confocal microscopes. eGFP was excited at 488 nm, while tRFP was excited at 561 nm. Image processing was performed using ImageJ/FIJI, while figure panels were assembled with Adobe Photoshop.

## Accession numbers

*BTR1*: AT5G04430; *DRB2*: AT2G28380; *DRB4*: AT3G62800; *HSP70*: AT3G12580; *HSP70-1* (or *HSC70-1*): AT5G02500; *HSP70-3* (or *HSC70-3*): AT3G09440; *NFD2*: AT1G24450; *RP27a*: AT1G23410; *RUP1*: AT3G45570; TRV RdRP: Q9J942; TRV CP: Q88897; and TRV 16k: Q77JX3.

## Supplemental data

The following materials are available in the online version of this article.

**Supplemental Figure S1**. Validation of B2 VSR activity and characterization of B2 Arabidopsis lines.

**Supplemental Figure S2**. Immunoblot validation of immunoprecipitates analyzed by MS.

**Supplemental Figure S3**. MS data analysis using additional controls. Supplemental Figure S4: lower magnification microscopy of samples in Figure 5.

**Supplemental Figure S5**. Analysis of ubiquitin detected by MS. Supplemental Figure S6: lower magnification microscopy of samples in Figure 6.

**Supplemental Figure S7**. Validation of T-DNA mutants.

**Supplemental Figure S8**. TRV accumulation in inoculated leaves.

**Supplemental Figure S9**. TRV accumulation in two *drb2* mutants.

**Supplemental Table S1**. List of primers and probes used in this study.

**Supplemental Data Set S1**. List of proteins detected by MS in GFP pull-downs from 35S:GFP/Col-0 and 35S:B2:GFP/Col-0 plants infected with TRV-PDS.

**Supplemental Data Set S2**. List of proteins detected by MS in GFP pull-downs from 35S:GFP/Col-0 and 35S:B2:GFP/Col-0 plants in both noninfected and TRV-infected conditions.

## Supplementary Material

koab214_Supplementary_Data

## Notes

Study conception and design: M.I. and C.R.; generation of transgenic *A. thaliana* lines: B.M.; genotyping: M.I. and E.V.; immunoprecipitation experiments: M.I.; RNA and protein extraction, northern and north-western blotting: M.I. and M.C.; western blotting: M.I., M.C. and H.S.; mass spectrometry: L.K.; statistical analysis of CoIP data: L.K. and H.S.; molecular cloning: M.I., B.M. and V.P.; recombinant B2 production: V.P.; plant inoculation and infection: M.I., M.C. and E.V.; laser confocal microscopy: M.I.; data analysis: M.I., M.C., B.M., L.K., H.S., V.P., P.D. P.G., C.R. writing: M.I. and C.R.; supervision: C.R.; and funding acquisition: C.R., P.G., and P.D.

The authors responsible for distribution of materials integral to the findings presented in this article in accordance with the policy described in the Instructions for Authors (https://academic.oup.com/plcell) are: Marco Incarbone (marco.incarbone@gmi.oeaw.ac.at) and Christophe Ritzenthaler (ritzenth@unistra.fr).
