# Supplementary material for: Immunocapture of dsRNA-bound proteins provides insight into Tobacco rattle virus replication complexes and reveals Arabidopsis DRB2 to be a wide-spectrum antiviral effector
Source: Plant Cell. 2021 Aug 26;33(11):3402–20. doi: 10.1093/plcell/koab214 (PMC8566308; doi:10.1093/plcell/koab214)
Supplement: koab214_Supplementary_Data [file koab214_supplementary_data.zip › tpc.20.00989_Supplemental Figures and Tables.pdf]

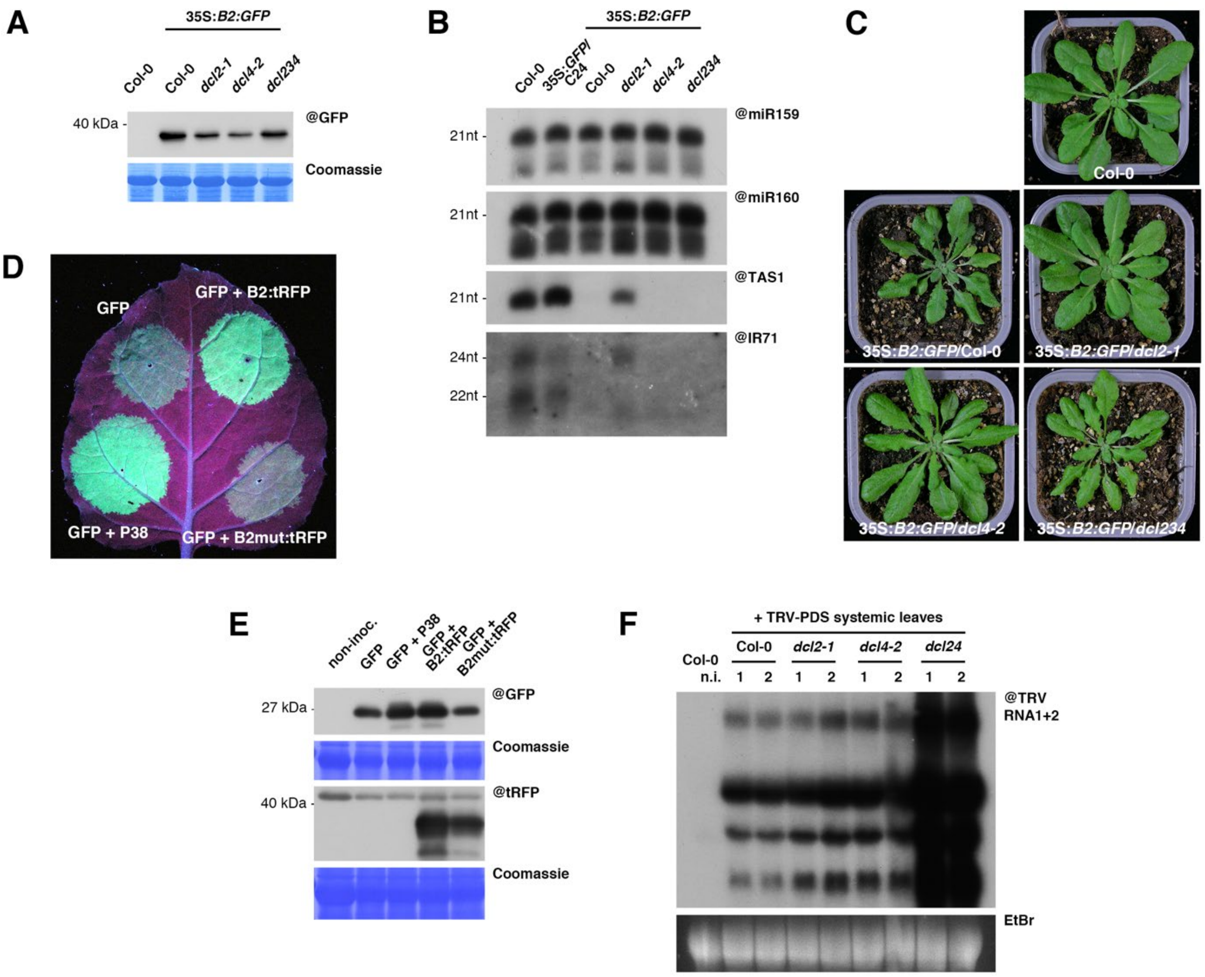

Supplemental Figure S1: validation of B2 VSR activity and characterization of B2 Arabidopsis lines. Supports Figure 1. **(A)** Immunoblot analysis to detect GFP in protein extracts from 35S:*B2:GFP* transgenic *A. thaliana* lines in Col-0 and *dcl2-1*, *dcl4-2* and triple *dcl2-1 dcl3-1 dcl4-2* mutants. Coomassie blue was used as loading control. **(B)** RNA gel blot analysis of low molecular weight RNA (PAGE gel) from the plants described in (A), to detect endogenous miRNA (miR159, miR160) and siRNA (TAS1, IR71). Probes were hybridized to the membrane through sequential stripping and probing. **(C)** Photos of the plants described in (A). **(D)** *N. benthamiana* leaf infiltrated with *A. tumefaciens* expressing GFP alone (top left) or in combination with P38, B2:tRFP or B2mut:tRFP, and illuminated with UV light. **(E)** Immunoblot analysis to detect GFP (top) and tRFP (bottom) in protein extracts from the infiltrated patches described in (E). **(F)** RNA gel analysis of high molecular weight RNA (agarose gel) from TRV-PDS systemically infected Col-0 and *dcl* mutant backgrounds. Source data is available at https://doi.org/10.5281/zenodo.5159940

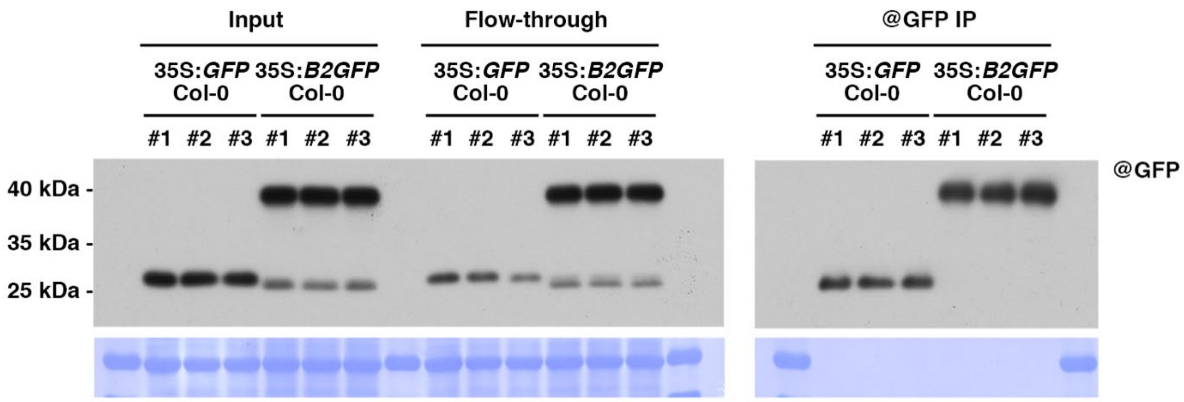

Supplemental Figure S2: immunoblot validation of immunoprecipitates analyzed by mass spectrometry. Supports Figure 2. Immunoblot analysis to detect GFP in protein extracts from the input, flow-through and anti-GFP immunoprecipitated fractions obtained from TRV-PDS-infected 35S:*GFP*/Col-0 and 35S:*B2:GFP*/Col-0 plants, performed in three technical replicates. Coomassie staining was used as loading control. The proteins from the immunoprecipitated fraction were further analyzed by mass spectrometry, and the results are shown in Supplemental Data Set S1. Source data is available at https://doi.org/10.5281/zenodo.5159940

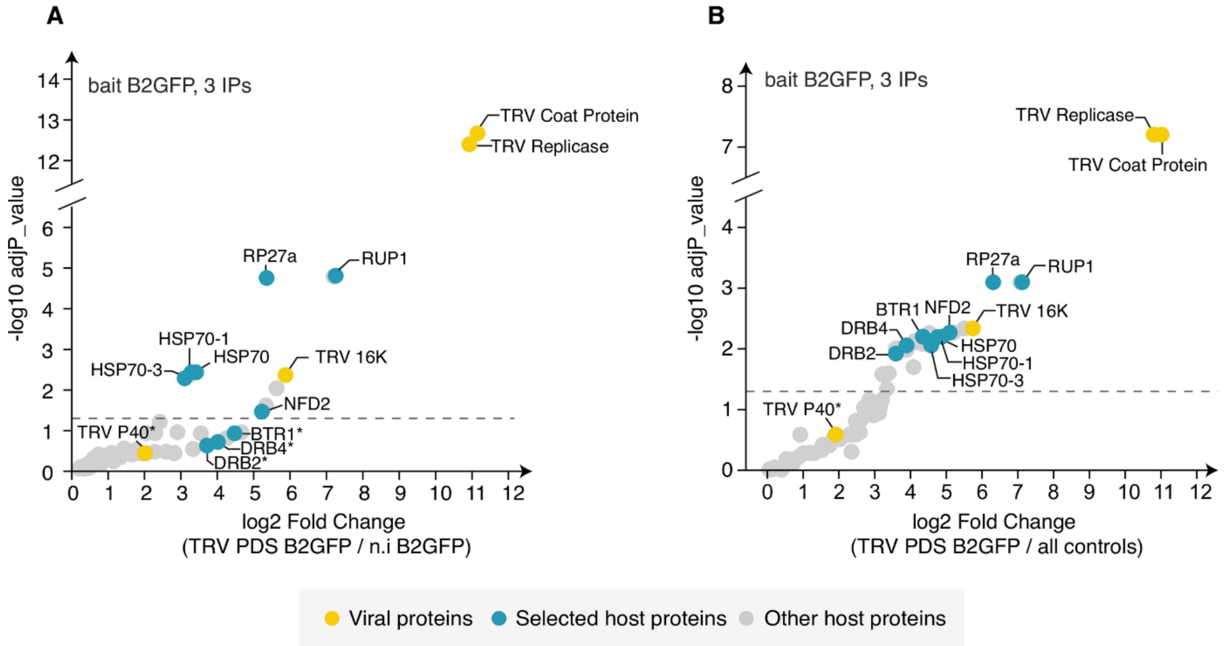

Supplemental Figure S3: mass spectrometry data analysis using additional controls. Supports Figure 2. Volcano plot representation showing the enrichment of proteins upon IP from TRV-infected 35S:*B2:GFP* Col-0 plants versus healthy 35S:*B2:GFP* Col-0 plants (**A**) and versus all controls: non-infected 35S:*GFP*/Col-0, non-infected 35S:*B2:GFP*/Col-0 and TRV-infected 35S:*GFP*/Col-0 (**B**). Y- and X-axis display adjusted p-values and fold changes, respectively. The dashed line indicates the threshold above which proteins are significantly enriched (adjP < 0.05). Selected proteins below significance level are indicated with a star. The source data are available in Supplemental Data Set S2.

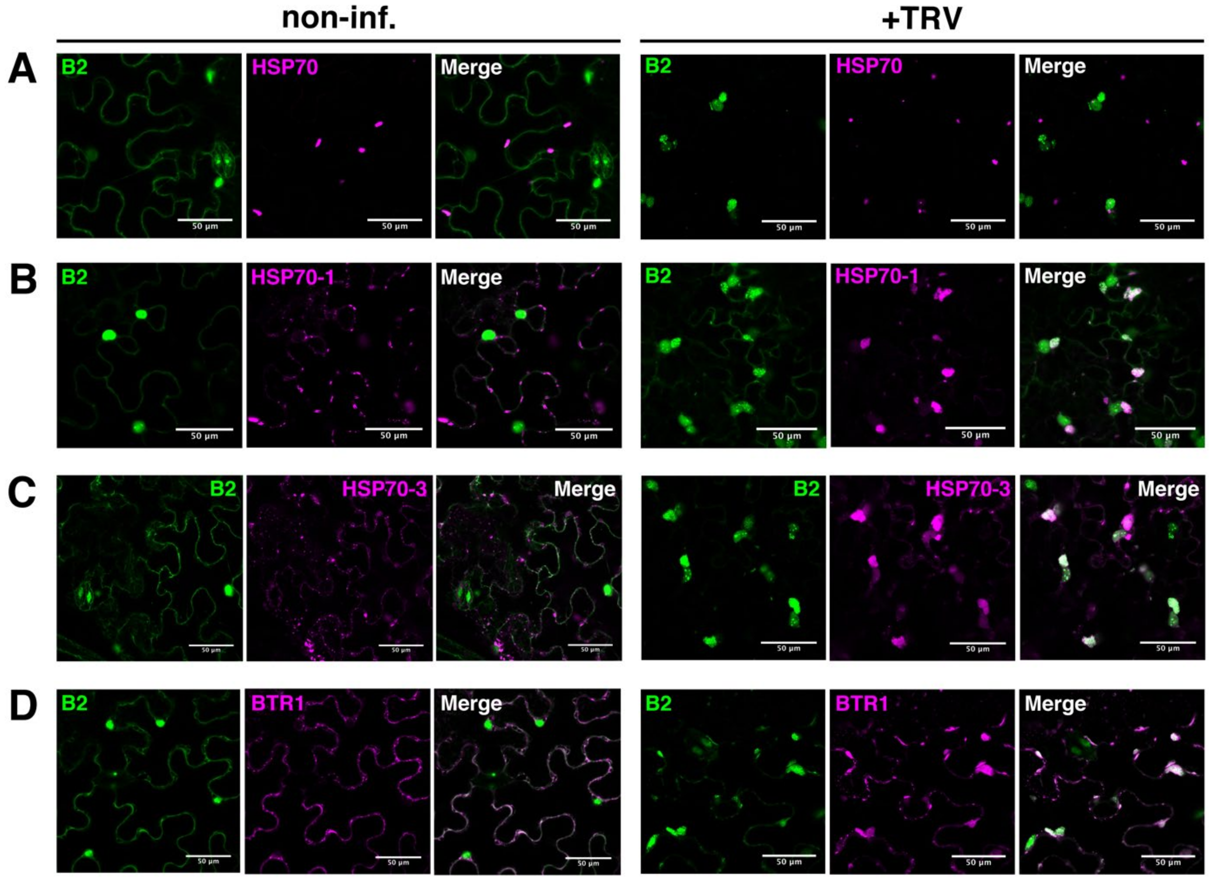

Supplemental Figure S4: lower magnification microscopy of samples in Figure 5. Supports Figure 5. Laser confocal microscopy (20x objective) on 35S:*B2:GFP/N. benthamiana* non-infected (left) and TRV-PDS-infected (right) leaf disks transiently expressing **(A)** 35S:*HSP70:tRFP,* **(B)** 35S:*HSP70-1:tRFP*, **(C)** 35S:*HSP70-3:tRFP*, **(D)** 35S:*BTR1:tRFP*. Scale bars indicate 50 µm. Additional acquisitions can be found with the Microscopy Source Data at https://doi.org/10.5281/zenodo.5159940

A

>sp|P59271|R27AA_ARATH Ubiquitin-40S ribosomal protein S27a-1 (RPS27AA)

MQIFVKTLTGKTITLEVESSDTIDNVKAKIQDKEGIPPDQQRLIFAGKQLEDGRTLADYNIQKESTLHLVLR

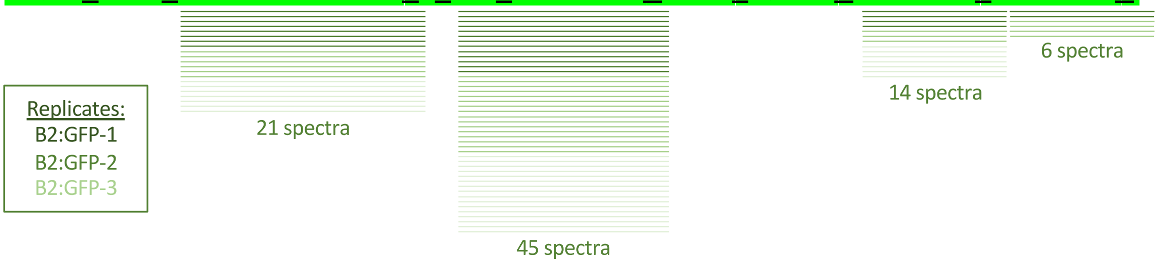

LRGGAKKRKKKTYTKPKKIKHTHKKVKLAVLQFYKVDGSGKVQRLKKECPSVSCGPGTFMASHFDRHYC
GKCGTTYVFKKADEE

B

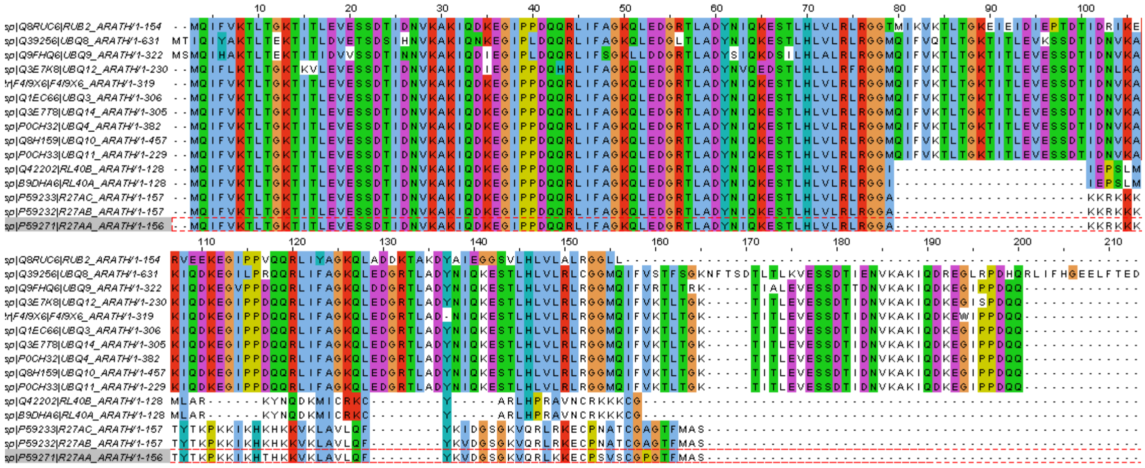

C

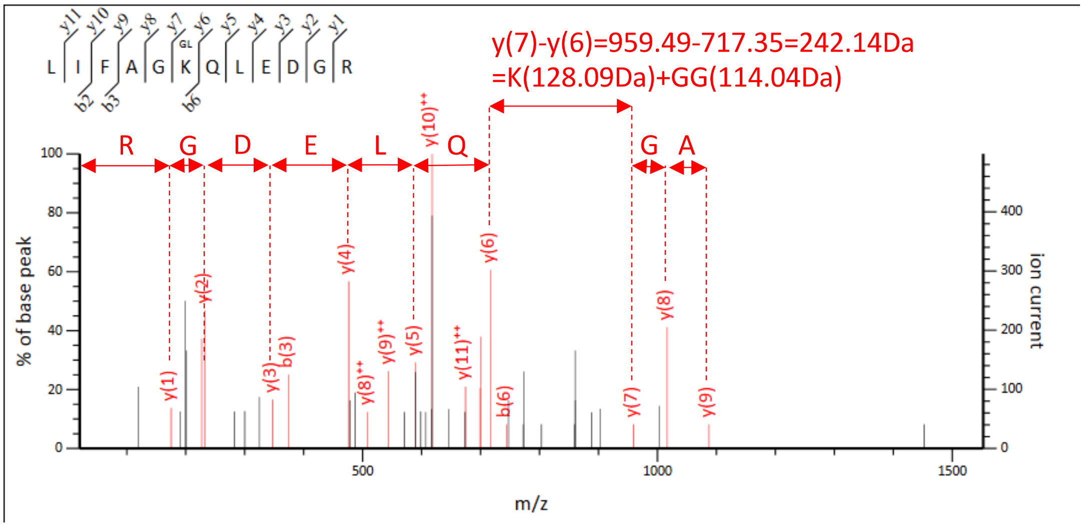

Supplemental Figure S5: analysis of ubiquitin detected by mass spectrometry. Supports Figure 2. **(A)** Sequence coverage obtained by MS on the RP27a protein (P59271). Four distinct tryptic peptides have been validated by Mascot algorithm at FDR<1% (bold residues). The total number of spectra matching on the 4 peptides are displayed as green bars with a color code according to the replicate. The Ubiquitin domain [1-76] is highlighted in green. Tryptic cleavage sites (K/R) are underlined. **(B)**

Multiple sequence alignment between RP27a sequence and UBQ1 to UBQ14 *A.thaliana* sequences (UniProtKB). Output generated with the MUSCLE tool (https://www.ebi.ac.uk/Tools/msa/muscle). **(C)** MS/MS spectrum corresponding to the ubiquitinated peptide [43-54] LIFAGK(Ub)QLEDGR identified by Mascot algorithm on RP27a protein (Score = 55.68, m/z=487.60, 3+, RT=42.97min). The fragmentation pattern involving the y- and b-ions validated by Mascot is displayed on the upper left corner. The di-glycine motif is highlighted by the GL abbreviation above the K-48 residue, as well as the mass difference between y(6) and y(7) fragments.

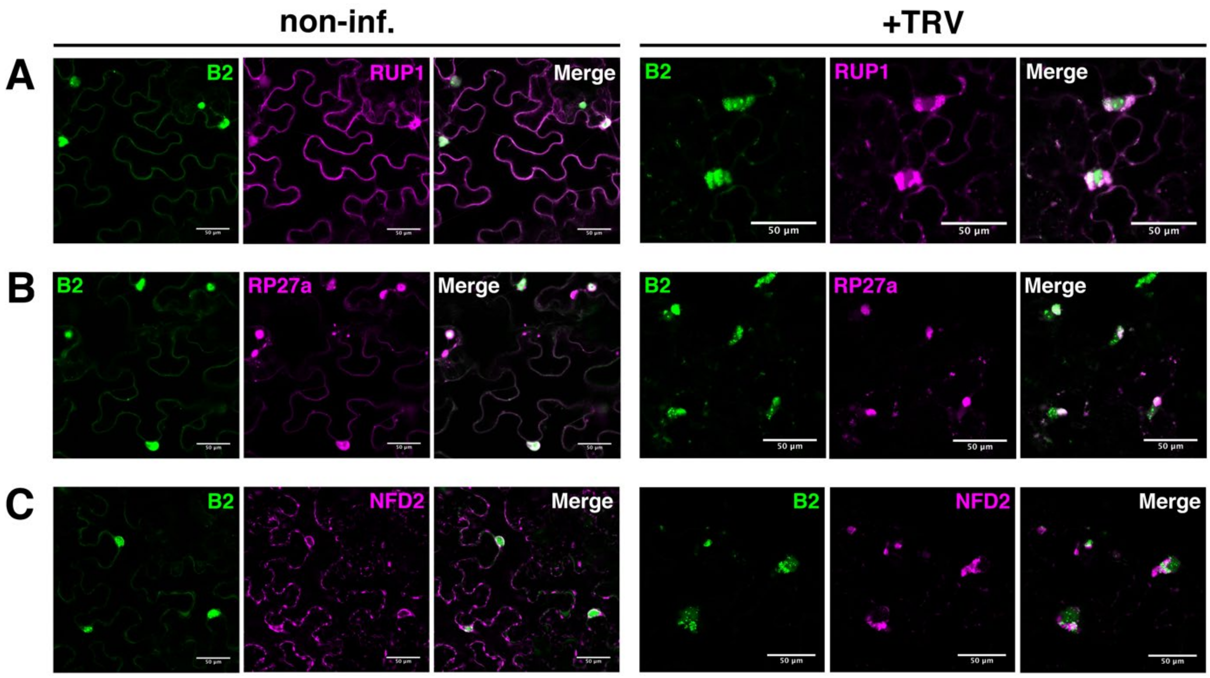

Supplemental Figure S6: lower magnification microscopy of samples in Figure 6. Supports Figure 6. Laser confocal microscopy (20x objective) on 35S:*B2:GFP/N. benthamiana* non-infected (left) and TRV-PDS-infected (right) leaf disks transiently expressing **(A)** 35S:*RUP1:tRFP,* **(B)** 35S:*tRFP:RP27a*, **(C)** 35S:*tRFP:NFD2*. Scale bars indicate 50 µm. Additional acquisitions can be found with the Microscopy Source Data at https://doi.org/10.5281/zenodo.5159940

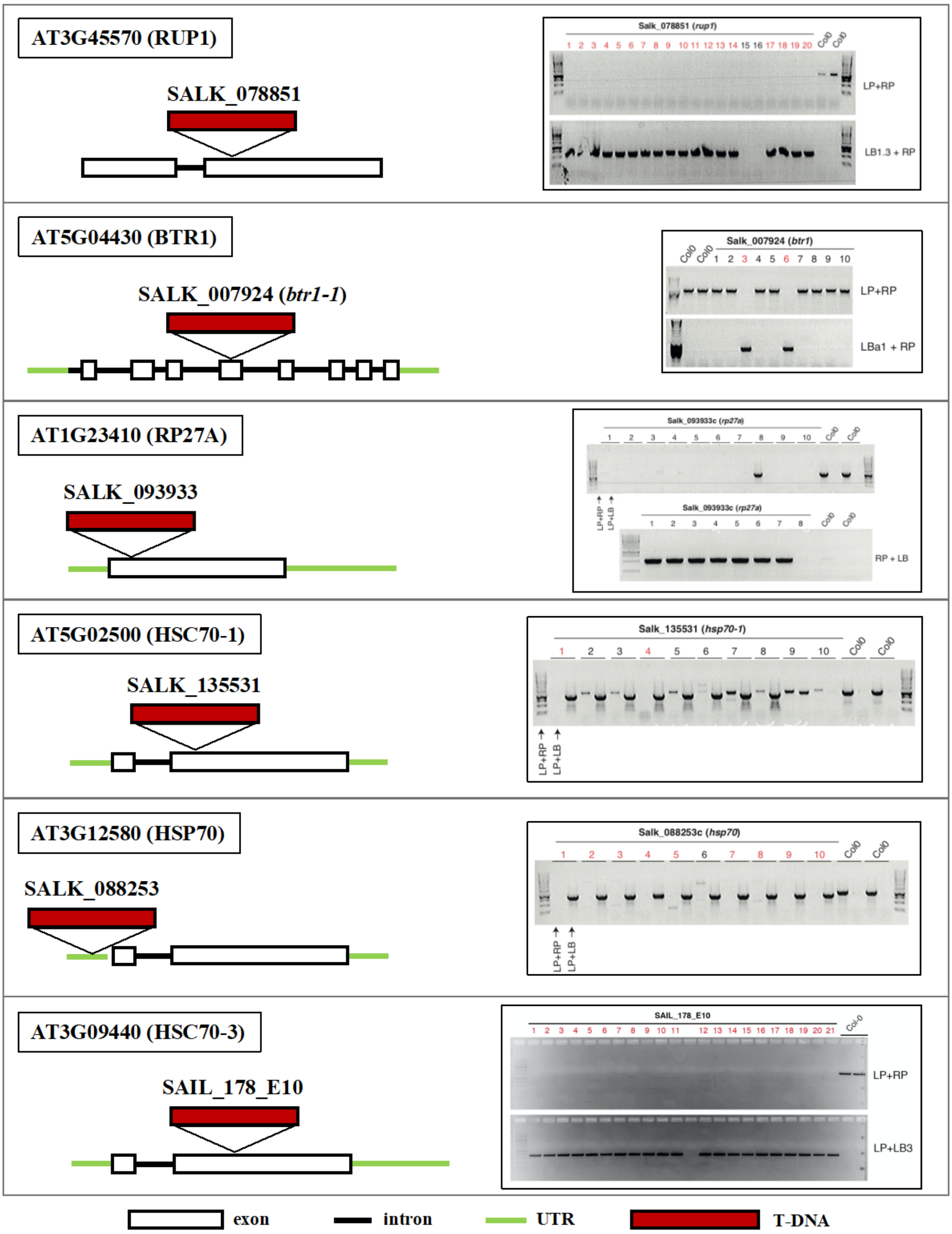

Supplemental Figure S7: validation of T-DNA mutant lines. Supports Figure 7. Description of the mutant T-DNA lines used in this work, except for the *dcl* and *drb* mutants, which are well-described in literature. Left: schematic representation of the T-DNA insertion loci within the respective gene products in the SALK/SAIL mutants analyzed, with a legend of the RNA transcript features annotated on TAIR (bottom). Right: PCR validation of these insertions. For all the lines (except SAIL_178_E10), plants carrying the homozygous mutant allele (red numbers above) were allowed to self-pollinate and set seed. The progeny of these plants was used for infection

experiments. In the case of SAIL_178_E10 (bottom) the plants used for genotyping were directly used in the infection experiment.

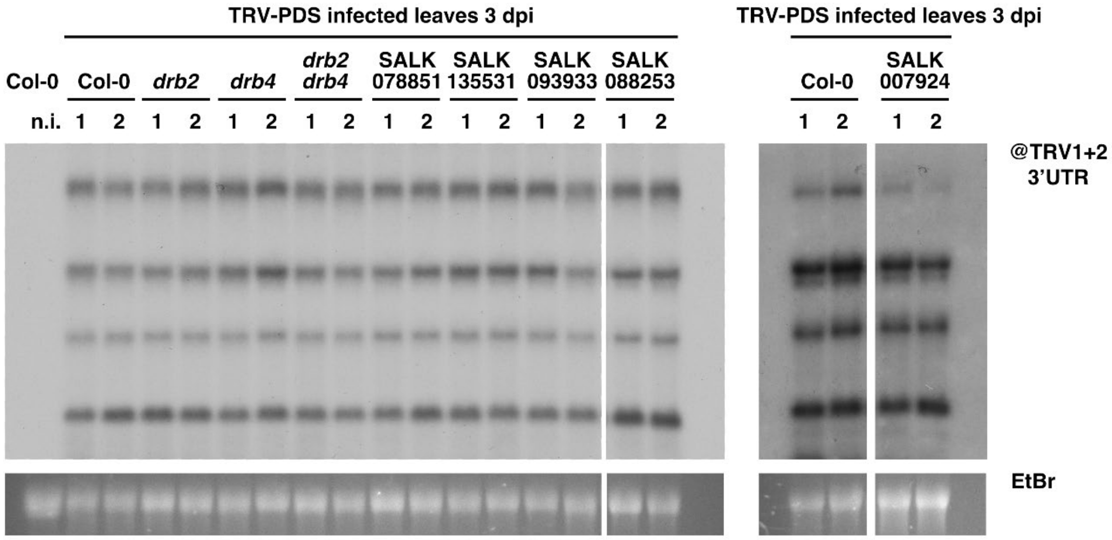

Supplemental Figure S8: TRV accumulation in inoculated leaves. Supports Figure 7. RNA gel blot analysis of RNA from inoculated leaves of Arabidopsis knock-out lines infected with TRV-PDS, 3 days post-infection (dpi). Previously published mutants are indicated with their current name, while the others are indicated with their SALK/SAIL nomenclature. Each sample is a pool of 4-5 plants (4 leaves per plant), and two samples were analyzed per genotype (1 and 2), per time point. EtBr staining was used as loading control.

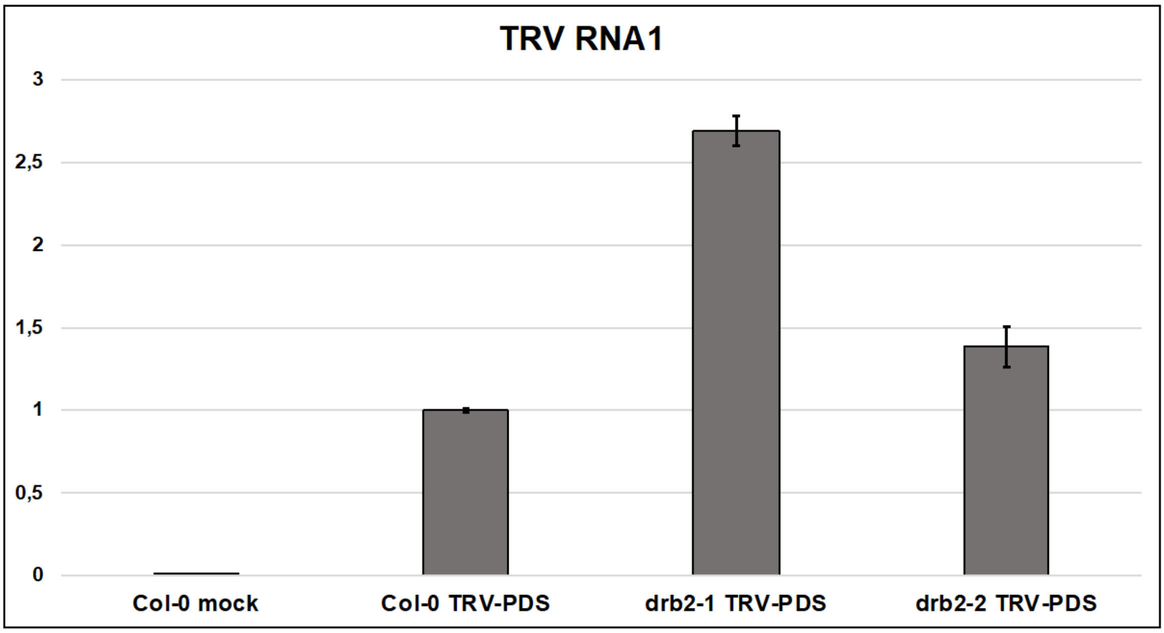

Supplemental Figure S9: TRV accumulation in two drb2 mutant lines. Supports Figure 7. Quantification of TRV RNA1 by RT-qPCR in systemic tissues of Col-0, *drb2-1* and *drb2-2* mutant lines. Each sample is a pool of systemically infected tissues from 12 plants. *AtSAND* and *AtGAPDH* were used as controls to normalize TRV1 accumulation.

Supplemental Table S1.
List of primers and probes used in this study.

Primer	Sequence
**Cloning**	
595 - BM_MutSapI-pET22>	CAGGCGCaCTTCCGCTTCCT
596 - BM_pET22-3086<	ATGCGGTATTTTCTCCTTACGCATCTGTG
638 - BM_pEAQ-MutNptII-1SapI<	cctgatgTtcCtcgtccagatcatcct
639 - BM_NptII-GLAPA>	ggctcgcgccagccga
631 - BM_MutB2-K47A>	AAACgcGGCAAAACTAACGGTAAGTC
632 - BM_MutB2-C44S<	AGGCtAGCGTGCAGGTTGT
640 - BM_pEAQ-MutNptII-2SapI<	cgccaagTtcCtcagcaatatcacg
641 - BM_NptII-GEWAD>	gcgaatgggctgaccgctt
589 - BM_XhoI-Sap3-K7ccdB<	CGATTCTCGAGTTATCACGAAGAGCTGTGTATAAGGGAGCCTGACA
642 - BM_AgeI-Sap1-K7ccdB>	tcACCGGTatggGAAGAGCTTAGGCACCCCAGGCTT
GG-NterRFP+	TATAGCTCTTCGATGagcgagctgattaaggag
GG-NterRFP-	TATAGCTCTTCCGCCTCCTCCTCCAGATCCTCCTCCattgtgccccagtttgctag
GG-CterRFP+	TATAGCTCTTCGTCCGGAGGAGGATCTGGAGGAGGAagcgagctgattaaggag
GG-CterRFP-	TATAGCTCTTCCTCAattgtgccccagtttgctag
GG-RFP-SKL-	TATAGCTCTTCCTCACAGTTTAGAattgtgccccagtttgctag
GG-CtagDRB2+	TATAGCTCTTCGATGTATAAGAACCAGCTACAAGAG
GG-CtagDRB2-	TATAGCTCTTCCGGAGATCTTTAGGTTCTCCAGTCG
DRB4nosap+	TCAAATGTGAAAAGCAGTCCAC
DRB4nosap-	GTGGACTGCTTTTCACATTTGA
GG-CtagDRB4+	TATAGCTCTTCGATGGATCATGTATACAAAGGTCAAC
GG-CtagDRB4-	TATAGCTCTTCCGGATGGCTTCACAAGACGATAGGC
GG-NtagNFD2+	TATAGCTCTTCGGGCATGGCGACTCTTCGTTTCAC
GG-NtagNFD2-	TATAGCTCTTCCTCACAACATAGAGACTAGTCTTCCA
GG-CtagBTR1+ (no SapI)	TATAGCTCTTCGATGGAGTCTACTGAGTCATATGCGGCAGGTTCACCGGAGGAGCTGGCTAAGAGATC
GG-CtagBTR1-	TATAGCTCTTCCGGAATCGGTGGTTCTCTCTGTG

GG-NtagRP27+	TATAGCTCTTCGGGCATGCAGATCTTCGTGAAAACC
GG-NtagRP27-	TATAGCTCTTCCTCACTCTTCATCAGCCTTCTTGAA
GG-CtagRUP+	TATAGCTCTTCGATGGAAGATGATCCAAAACCC
GG-CtagRUP-	TATAGCTCTTCCGGATATATCTGCTGTAGGATTTGGACC
GG-CtagH70+	TATAGCTCTTCGATGGCGGGTAAAGGTGAAG
GG-CtagH70-	TATAGCTCTTCCGGAATCAACTTCTTCAATCTTTGGGC
GG-CtagH70-1+	TATAGCTCTTCGATGTCGGGTAAAGGAGAAGG
GG-CtagH70-1-	TATAGCTCTTCCGGAGTCGACCTCCTCGATCTTA
GG-Ctag H70-3+	GGTGGTGCTCTTCGATGGCTGGTAAAGGAGAAGG
GG-Ctag H70-3-	ACCACCGCTCTTCCGGAGTCGACTTCCTCAATCTTGGG
**Probes:**	
Oligos:	
TRV 1+2 3'UTR (oligo probe for high molecular weight northern blot)	GCCTTTGTAACCATCATCACT
U6	AGGGGCCATGCTAATCTTCTC
miR159	TAGAGCTCCCTTCAATCCAAA
miR160	TGGCATACAGGGAGCCAGGCA
TBSV 3'probe	GGGCTGCATTTCTGCAATGTTCC
Primers for PCR (Klenow labeling)	
PDS+	AGATTTGACTTCCCAGATGTC
PDS-	ACCATATATGAACATTAATAACTG
Primers for genotyping	
SALK_078851 LP	TTTTTCGGAGAGATAATCTCCG
SALK_078851 RP	TTGATACAAAAGGGTTCACCG
SALK_093933 LP	CTTAACTCCAGATTTTCCCGC
SALK_093933 RP	CACATTTCCCACAATAATGCC
SALK_088253 LP	CCAAATACGAAGCCACTTGAG
SALK_088253 RP	TACCGAAGACGGTGTTGGTAG
SALK_135531 LP	AAGGAGAAGGACCAGCTATCG
SALK_135531 RP	TCTTCGCTCTCTCACAGGAAG
SAIL_178_E10 LP	CCAGAAAGCTCGAATTTACCC
SAIL_178_E10 RP	TTGTGTGACAGATGCGAAGAG
SALK_007924 LP	CGGGATCAATCAAAGAAGTTG
SALK_007924 RP	CAAGGGATATGATCCAGCATC

LBb1.3 (SALK)	ATTTTGCCGATTTCGGAAC
LB3 (SAIL)	tagcatctgaatttcataaccaatctcgatacac
Primers for qPCR	
TRV1 qPCR +	TGCCAGCTGTCAAACCTTTG
TRV1 qPCR -	ACTGACAACCGGCCTTTTTG
AtGAPDH +	TTGGTGACAACAGGTCAAGCA
AtGAPDH -	AAACTTGTCGCTCAATGCAATC
AtSAND +	AACTCTATGCAGCATTTGATCCACT
AtSAND -	TGATTGCATATCTTTATCGCCATC
